# Supplementary material for: The effects of dabrafenib and/or trametinib treatment in Braf V600-mutant glioma: a systematic review and meta-analysis
Source: Neurosurg Rev. 2024 Aug 22;47(1):458. doi: 10.1007/s10143-024-02664-x (PMC11341626; doi:10.1007/s10143-024-02664-x)
Supplement: Supplementary file 1 — Supplementary file1 Supplementary Figure 1.Forest plot for PFS rate (a) and Subgroups analysis of PFS rate by the intervention (b). Supplementary Figure 2. Forest plot for PR (a) and CR (b). Supplementary Figure 3.Forest plot for ORR (a) and RR (b). Supplementary Figure 4. Subgroups analysis of RR by age (a) and by the intervention (b). Supplementary Figure 5. Subgroups analysis of AEs by age (a) and by the intervention (b). Supplementary Figure 6.The funnel plot for PFS. Supplementary Figure 7.The funnel plot for PFS rate. Supplementary Figure 8.The funnel plot for OS. Supplementary Figure 9.The funnel plot for PR. Supplementary Figure 10.The funnel plot for CR Supplementary Figure 11.The funnel plot for ORR. Supplementary Figure 12.The funnel plot for RR. Supplementary Figure 13.The funnel plot for AEs Supplementary Figure 14.The funnel plot for death events. Supplementary Figure 15.The funnel plot for PFS. Supplementary Figure 16.The funnel plot for PFS rate. Supplementary Figure 17.The funnel plot for OS Supplementary Figure 18.The funnel plot for PR. Supplementary Figure 19.The funnel plot for CR. Supplementary Figure 20.The funnel plot for ORR. Supplementary Figure 21.Sensitivity analysis of RR. Supplementary Figure 22.Sensitivity analysis of AEs. Supplementary Figure 23.Sensitivity analysis of death events (ZIP 1916 KB) [file 10143_2024_2664_MOESM1_ESM.zip › Manuscript-tracked.docx]

**The Effects of Dabrafenib and/or Trametinib treatment in BRAF V600-Mutant** **Glioma: a systematic review and meta-analysis**

**Running title:** Dabrafenib combined with trametinib in BRAF v600-mutant PLGG

Jun Lei, Yanhui Liu*, Yingjun Fan

Department of Neurosurgery, West China Hospital, Sichuan University, No.37 Guoxue Lane, Wuhou District, Chengdu 610041,China.

*Corresponding author:

Yanhui Liu

E-mail: [liuyh@scu.edu.cn](mailto:liuyh@scu.edu.cn)

TEL: +86-15884463100

Word count: 2700

Figure count:3

Table count: 2

**Abstract**

**Objective:** This study aimed to evaluate the effects of dabrafenib and/or trametinib therapy in BRAF v600-mutant glioma treatment.

**Methods**: PubMed, the Cochrane Library, EMBASE and Web of Science were searched from inception to Sep 2023. Inclusion criteria were designed based on the PICO principle to select relevant articles. Search keywords included *‘dabrafenib’, ‘trametinib’, ‘glioma’* and other related keywords. Outcomes included overall survival (OS), progression-free survival (PFS), adverse events (AEs), and death events. Methodological index for non-randomized studies (MINORS) was used to assess the methodological quality. Stata 14.0 was selected to perform the Cochrane Q and I^2^ statistics to test the heterogeneity among all studies. As for publication bias assessment and [sensitivity analysis](javascript:;), the funnel plot, Egger regression test, Begg test, and trim and fill method were selected.

**Results:** Including 8 studies for meta-analysis. The pooled results of the single-arm trials showed that the median PFS and median OS after treatment were 6.10 months and 22.73 months, respectively. Notably, this study found a high incidence of AEs and death events of 50% and 43% after treatment. All the above findings were statistically significant. Also, this study statistically supported the advantage of disease response improvement after the combination therapy in BRAF v600-mutant glioma patients, which were shown as a pooled rate of PR (30%), a pooled rate of CR (18%), and a pooled rate of ORR (39%). And the AE rate was lower in the monotherapy group (AE: 25%) than in the combination treatment group (AE: 60%). Sensitivity analysis indicated that all the results were robust.

**Conclusions:** Based on current literature outcomes, dabrafenib and/or trametinib may lead to the median PFS of 6.10 months and median OS as 22.73 months for BRAF v600-mutant glioma patients, and the safety of monotherapy is better than that of combination therapy. This conclusion needs to be treated with caution and further verified.

**Keywords:** dabrafenib, trametinib, pediatric BRAF v600-mutant, glioma, meta-analysis

**Introduction**

Gliomas are intrinsic brain tumors derived from glial progenitor cells [1]. As the most common primary malignant brain tumors in adults, gliomas occur primarily in the brain and glial tissue and are usually malignant [2].[3, 4] As a large histological category, there is no consistent definition of glioma, and its incidence varies according to age, histologic type, sex, and ethnicity [5]. Among adults, glioblastoma is the subtype with the highest prevalence and mortality, with an age-adjusted incidence of 0.59 to 3.69 per 100,000 [6] [7]. On the contrary, the prognosis of pediatric low-grade glioma (PLGG) was pretty good, with a 10-year overall survival reaching a maximum of 96% [8]. [4, 8, 9]During the follow-up, complete surgical resection is the primary treatment to be considered once tumor progression or some symptoms occur, and the postoperative long-term prognosis is usually excellent. However, in cases where complete surgical resection cannot be accepted, such as the lesion area involving the optic nerve crossings or pathways, or tumor progression occurs despite resection, adjuvant therapies such as irradiation and chemotherapy are selected [10]. However, according to several studies, it had been shown [4, 8, 10, 11] that irradiation was associated with medium- and long-term neurological toxicity. Chemotherapy, in addition to its non-negligible adverse effects, meant that the frequency of attendance to the outpatient increased, which invariably raised the cost of time. Therefore, searching for more and better treatments is crucial to achieving long-term disease control in glioma. In recent decades, there has been a great deal of interest in the key genes that drive cancer, and searching for therapeutic targets at the genetic aspect is a popular trend in the field of oncology nowadays. The B-Raf proto-oncogene serine / threonine-protein (BRAF) V600E mutation has been focused on by researchers as a potential oncogenic factor in many types of cancer [12-14], such as papillary thyroid cancer, colorectal cancer, melanoma, and gliomas in both adult and pediatric populations. It has been shown that the BRAF V600E mutation was found in nearly 20% of low-grade glioma patients [15-17] and this alteration may play an oncogenic role by constitutively activating the mitogen-activated protein kinase (MAPK) signaling pathway (also known as the RAS/RAF/MEK/ERK pathway). In addition, some studies [17, 18]concluded that there was a strong association between BRAF V600 mutant and the transformation of low-grade glioma (LGG) into secondary high-grade glioma (HGG). Therefore, in recent decades, a lot of researchers [19-21] have paid attention to the therapeutic roles of BRAF inhibitors and MEK inhibitors (a key inhibitor of the MAPK pathway) in BRAF V600E mutant glioma.

Dabrafenib, a BRAF inhibitor, acts by selectively inhibiting mutant BRAF kinases but is prone to acquired resistance, which can be mitigated if combined with trametinib (which exerts inhibitory effects on MEK1, MEK2 and kinase activity) [22, 23]. Meanwhile, Trametinib, as a MEK1/2 inhibitor, has also been shown in some studies to produce safe and effective anti-glioma effects by monotherapy or combination [24, 25]. Moreover, studies conformed that the combination of trametinib and dabrafenib can block the MAPK pathway through dual inhibition, inhibit the production and survival of brafv600 mutant cells, and enhance the anti-tumor activity, thus exerting therapeutic advantages [26]. Clinically, dabrafenib combined with trametinib had shown good prolongation of overall survival (OS) and progression-free survival (PFS) in adult patients with CNS tumors compared to single drug [27-29], laying the basis for the use of dabrafenib combined with trametinib in pediatric patients. In 2023, dabrafenib and trametinib [30]were approved for use in pediatric patients by the U.S. Food and Drug Administration (FDA) and were recommended for the treatment of LGG patients over 1 year old with the BRAF V600E mutation who required systemic therapy. Over few years, there had occurred several meta-analyses on the effects of dabrafenib combined with trametinib in unresectable or metastatic melanoma, metastatic or advanced non-small cell lung cancer with BRAF V600E mutation [31, 32]. There is also some accumulation of evidence in the field of LGG treatment, with controlled studies showing that the objective response rate (ORR) of combination therapy (25%) was better than that of monotherapy (15%).[33] However, to the best of our knowledge, there are currently no meta-analyses summarizing the published evidence involving the efficacy of dabrafenib and/or trametinib treatment in BRAF V600E mutant glioma patients.. Therefore, this meta-analysis aimed to explore the effects of dabrafenib combined with trametinib treatment in BRAF V600-Mutant glioma patients by comprehensively analyzing currently available studies.

**Material and methods**

The present systematic review with meta-analysis was conducted according to the Preferred Reporting Items for Systematic Reviews and Meta-Analyses (PRISMA 2020) guideline [34]. This study is registered with the PROSPERO registry, number CRD42024518699.

Search strategy

PubMed, the Cochrane Library, EMBASE and the Web of Science database were comprehensively searched for relevant trials from their inception until Sep 2023. This study picked up the medical subject heading (MeSH) term of ‘dabrafenib’ ‘dabrafenib mesilate’ ‘trametinib’ ‘gsk 1120212’ ‘glioma’ and ‘gliomas’ and other keywords to perform the search strategy. The detailed search strategy is placed in **Table S1.**

Inclusion and exclusion criteria

Inclusion and exclusion criteria in the present study were based on the Population, Intervention, Comparator, Outcomes, and Study designs (PICOS) structure.

1. Population: studies included participants with BRAF V600 mutation-positive glioma.
2. Intervention: participants received dabrafenib and/or trametinib treatment.
3. Comparator: no restrictions on the intervention methods of the control group.
4. Outcome: studies provided progression-free survival (PFS), overall survival (OS), adverse event(AEs), PFS rate, partial response (PR), complete response (CR), objective response rate (ORR), or response rate (RR) (including partial response, complete response, and minor response) as outcomes.
5. Study design: studies with any comparative designs, or single-arm observational designs.

Besides, conference abstracts, case reports, reviews, studies with incomplete data, and repeated reports of the same study were excluded.

Data extraction

Available studies were selected by two authors independently, which included screening abstracts and titles and checking full texts. Disagreements between them were resolved by a third one. The following information were extracted from included studies: publication year, author’s name, country, sample size, study design, age, clinical diagnosis, female proportion, interventions of the experimental group or controlled group, and outcomes.

Quality assessment

Methodological index for non-randomized studies (MINORS) [35] was used to assess the methodological quality of all observational studies. This tool consisted of 8 criteria for all studies and 4 added criteria specifically for controlled studies. Each criterion was scored 0, 1, or 2 (where 0 showed high risk, 1 showed unclear risk, and 2 showed low risk), and the sum for each study was calculated.

Statistical analysis

The meta-analysis was conducted using STATA 14.0 (StataCorp, College Station, Texas, USA). Pooled effects of PFS, PFS rate, OS, PR, CR, ORR, RR and AEs were calculated. If the 95% CIs of the rates exceeded 100%, the “metaprop” command was used; otherwise, the “metan” command was used [36]. The study used I-squared (I2) and χ2 to evaluate the heterogeneity. The random-effect model was adopted if the p≤0.10 and I2 ≥50%, which meant existing heterogeneity among studies model. Otherwise, the fixed-effect model was applied. Publication bias was assessed using funnel plots, the Begg rank correlation [37] and Egger weighted regression [38]. If significant bias was present, trim-and-fill analysis was used to judge whether the publication bias had an impact on the outcomes. Subgroup analysis was conducted to explore possible sources of heterogeneity in different age (>18 or ≤18), interventions (dabrafenib and trametinib, dabrafenib or trametinib), and clinical diagnosis (HGGs and/or LGGs, PHGG, PLGG).

Subgroup analysis was performed to explore possible sources of heterogeneity if necessary. Sensitivity analysis by leave-one-out method was used to test the robustness of the results. P < 0.05 indicated statistical significance.

**Results**

Study selection

To sum up, a total of 720 studies were retrieved as potentially relevant literature reports through the initial searches. Among these studies, 81 records were marked as duplicates by automation tools, and 232 records were excluded after reviewing the title and abstract since they were not related to the topic of this research article. After excluding 397 inaccessible and unavailable studies, 10 studies remained for full-text screening. There were 2 studies without full-text, and 8 studies [10, 19-21, 29, 33, 39, 40] were eligible for our analysis. The flow chart is in **Figure 1.**

Study characteristics

The eight trials that met the inclusion criteria were published between 2021 and 2023, with sample sizes ranging from 5 to 58. The eight studies included two controlled trials and six single-arm trials. The studies were conducted in one each in Spain, the USA, England, and Canada. Most of the study population were children. Female proportion ranged from 47.83 to 56.1. The participants’ demographic characteristics were summarized in **Table 1.**

Quality assessment

Quality assessment was performed among each included study by MINORS. The six single-arm studies received 14,15 and 16 points, respectively. Major issues focused on possible bias in the evaluation of target outcomes, a loss of follow-up rate of more than 5% and no prospective calculation of the sample size. One of the two controlled studies received a perfect score [33], and the other trials had four alarming points, including loss to follow up exceeding 5%, no prospective calculation of the sample size, control group not having the gold standard intervention, and without baseline equivalence of groups [29]. The results of the included trials in this meta-analysis were at low risk (**Table 2**).

Primary Outcomes

Progression-free survival and overall survival

Three trials provided PFS as outcomes [19, 21, 40]. The meta-analysis showed that PFS after dabrafenib and trametinib treatment was 6.10 (95%CI: 3.09-9.11, I^2^= 0.0%, P=0.382) months (**Figure 2(a)**). Moreover, two studies presented OS [19, 21], dabrafenib combined with trametinib led to an OS of 22.73 (95%CI: 5.26-40.21, I^2^=90.2%, P=0.001) months (**Figure 2(b)**).

Adverse events (AEs) and death events

Seven studies presented data about AEs [20, 21, 29, 33, 40]. The pooled rate of AEs was 50% (95%CI: 9%-91%; I^2^=99.4%, P<0.001) (**Figure 3(a)**). However, the subgroup analysis showed the high heterogeneity of AEs may not result from the intervention, age, or clinical diagnosis (**Supplementary Figure 1**). However, the AE rate was lower in the monotherapy group (AE: 25%; 95%CI: -28%-78%; I^2^=93.4%) than in the combination treatment group (AE: 60%; 95%CI: -29%-90%; I^2^=97%). Moreover, the pooled rate of death events [21, 40]was 43% (95%CI: 26%-60%; I^2^=68.3%, P=0.076) (**Figure 3(b)**).

Secondary Outcomes

PFS rates

Three studies presented the PFS rates [10, 39, 40], and the pooled PFS rate was 79% (95%CI: 51%-107%, I^2^=96.3%, P<0.001) (**Supplementary Figure 2(a)**). Without heterogeneity reduction, no statistically significant difference in the PFS rates was found by intervention subgroup analysis (**Supplementary Figure 2(b)**).

Disease response

Two studies [20, 40]presented complete response (CR) and partial response (PR). The pooled rate of PR was 30% (95%CI: 21%-39%, I^2^=0.0%, P=0.827) (**Supplementary Figure 3(a)**) while the pooled rate of CR was 18% (95%CI: 6%-42%, I^2^=88.8%, P=0.003) (**Supplementary Figure 3(b)**).

Moreover, two studies [10, 40] presented objective response rate (ORR) and six studies presented response rate (RR) [10, 19, 21, 33, 39]. The pooled ORR was 39% (95%CI: 3%-31%; I^2^=93.3%, P<0.001) (**Supplementary Figure 4(a)**) while the pooled RR was 58% (95%CI: 26%-90%; I^2^=96.2%, P<0.001) (**Supplementary Figure 4(b)**). After the subgroup analysis of RR by different interventions, age, and clinical diagnosis, the heterogeneity was markedly decreased in the age subgroup of more than 18 years old patients (I^2^=0.0%, P=0.333), intervention subgroup of dabrafenib or trametinib (I^2^=32,1%, P=0.225), and clinical diagnosis subgroup of HGGs and/or LGGs patients (I^2^=42.1%, P=0.189) (**Supplementary Figure 5**).

Publication bias and Sensitivity analysis

The study used the funnel plot, and Begg and Egger's test to evaluate the publication bias. The funnel plots showed there may exist publication bias in PFS，PFS rate, OS, PR, CR, ORR, RR, AEs, and death events (**Supplementary Figure 6-14**), however this possibility was negated by Begg and Egger's test (P>0.05) (**Supplementary Table 2**). . However, the sensitivity analysis results and funnel plots found the existence of heterogeneity in PFS，PFS rate, OS, PR, CR, ORR, RR, AEs, and death events analysis, suggesting that analysis results should be treated with caution (**Supplementary Figure 15-23**).

**Discussion**

The meta-analysis, which included a comprehensive collection of 8 studies, revealed the effects of dabrafenib combined with trametinib in BRAF V600 mutation-positive glioma. This meta-analysis presented a PFS of 6.10 months, an OS of 22.73 months, a pooled AEs rate of 50% and a death events rate of 43%. All the above were statistically significant. Moreover, a pooled rate of PR was 30%, a pooled rate of CR was 18%, a pooled ORR was 39%, and a pooled RR was 58%. Significant heterogeneity was observed in OS, AEs, death event, PFS rates, CR, ORR, and RR analysis, with intervention, age, and clinical diagnosis was discovered as potential confounders for RR results.

PFS and OS are efficacy outcomes we generally discussed about oncological treatments, which are both intuitive and often calculated statistically. Our results showed that after the treatment of dabrafenib combined with trametinib, the PFS and OS of BRAF V600 mutation-positive glioma patients were 6.10 months and 22.73 months, respectively, which showed an anti-tumor activity and improvement of disease progression. Similar results were also found in several trials [19, 21, 40]. Nonetheless, most of trials included in this study were single-arm and were lacking in controlled studies with other therapies which were crucial for better selection and use of dabrafenib combined with trametinib in the future. A case report about recurrent BRAF V600E-mutant adult gliomas [41] found that after a failure of a BRAF inhibitor alone, a BRAF inhibitor combined with a MEK inhibitor could exert a markedly prolonging OS. Moreover, a phase 2 clinical trial [42] showed that 47% of patients treated with dabrafenib plus trametinib achieved an overall response, compared to 11% in the chemotherapy group. Additionally, the median progression-free survival was significantly longer in the dabrafenib plus trametinib group (20.1 months) compared to the chemotherapy group (7.4 months). In addition to the above advantages compared with either alone or standard chemotherapy, several studies [17, 18, 29] had found that a BRAF inhibitor combined with a MEK inhibitor had superior PFS and OS after radiotherapy, chemotherapy, or the clinical standard care surgery and was well tolerated. All the above studies suggested that clinical trials of rare diseases were mostly single-arm, and the lack of high-quality studies, such as RCTs, was common and inevitable nowadays. Therefore, if the number of relevant single-arm studies was sufficiently large in the future, we can consider to perform subgroup analyses to discuss respectively, such as patients with low-grade gliomas containing highly heterogeneous tumors with different prognoses, or patients with different surgical manages (no, partial or total tumor resection) [21, 43], then finding out the best suitable population for darafenib combined with trimetinib and making suggestions for the determination of the dosage regimen.

In addition to PFS and OS, disease response is also an indicator that has been focused on in tumor therapy. Our results showed that PR, CR, ORR, and RR of dabrafenib combined with trametinib in BRAF V600 mutation-positive glioma were, 30%, 18%, 39%, and 58%, respectively. Compared with single drug, Nobreet al [44] also supported the advantage of disease response of the combined therapy, with a rapid and long-lasting response, which was obtained within 2 months and sustained over 24 months. Moreover, the objective response rate of the combined therapy was more obvious in LGG compared to patients with BRAF V600E mutated HGG (ORR of 26%) [45].These studies reminded us that if there were enough controlled studies available for analysis in the future, the efficacy of the combined therapy versus (VS) single drug in LGG, including how soon to start responding and how long a response sustains, or the efficacy of the combined therapy in low-grade VS high-grade gliomas should be focused on. In addition, recently, a study [39] found that 20% of 70 patients with PLGG had inconsistent responses, and later, it was concluded that different assessments had their own tendencies and focus by performing a comparison of volumetric and 2D tumor assessments, which reminded us that we should identify a uniform assessment standard in subsequent oncology studies.

The occurrence of adverse events (AEs) and deaths during the use of novel targeted drugs also should be paid attention to. Lots of trials [10, 46, 47]have indicated that AEs associated with dabrafenib combined with trametinib are most common in the skin, gastrointestinal symptoms, or some severe AEs such as atrial fibrillation and pulmonary embolism. This study showed that after treated with dabrafenib combined with trametinib, the rates of AEs and death events in BRAF V600 mutation-positive glioma were 50% and 43%, respectively. It was a rate difficult to ignore. Such a high risk of AEs may be related to the reason that all adverse events were counted together in this study. In fact, AEs for dabrafenib combined with trametinib were generally mild-moderate symptoms, such as fever, headache, fatigue, and nausea, with low incidence rate of grade ≥3 AEs like ocular changes, cardiomyopathy, and pneumonia [48-50]. As for the high risk of death, some participants may had other potentially high mortality-risk primary conditions before, and it was difficult to separately perform subgroup analysis of these patients for the limited amount of available studies [51-53]. In conclusion, the findings should be treated with caution.

It is crucial to consider the limitations in this study. First, potential language bias might exist because only studies published in English were included. Second, the outcomes mentioned in this study did help us to evaluate the effects of dabrafenib combined with trametinib in BRAF V600 mutation-positive glioma to a great extent, but if there are more high-quality studies in the future, we may make a more comprehensive evaluation together with other more evaluation items, such as neurocognitive and psychological evaluation, which depended on a fact that BRAF V600 mutation-positive glioma survivors face long-term psychological and neurocognitive morbidities [39].Thirdly, the studies included in this study was limited. Most were single-arm, and only 2 controlled studies were included. The lack of a sufficient number of controlled studies made it difficult to get rid of the influence of confounding factors on the reliability of the results. Based on several studies encouraging a conventional molecular testing for BRAF mutation-targeted alterations in clinical diagnosis [19, 21], a large number of controlled studies that can be pooled and analyzed will undoubtedly emerge in the future. Therefore, we can continue to follow up in this aspect. Moreover, no publication bias existed in all results and sensitivity analysis indicated that the pooled effect size results were robust.

**Conclusions**

To sum up, dabrafenib combined with trametinib may have potential effects on improving survival, disease response, and AEs of patient with BRAF v600-mutant PLGG based on current literatures’outcomes, and highlighted the need for more high-quality studies.

**Ethics approval and consent to participate**

Not applicable.

**Funding**

None.

**Conflict of interests**

All authors declare that they have no conflict of interests.

**Author contributions**

Jun Lei carried out the studies, participated in collecting data, and drafted the manuscript. Yanhui Liu performed the statistical analysis and participated in its design. Yingjun Fan helped to draft the manuscript. All authors read and approved the final manuscript.

**Availability of data and materials**

All data generated or analysed during this study are included in this published article.

**References:**

1. S Xu, L Tang, X Li, F Fan, Z Liu (2020) Immunotherapy for glioma: Current management and future application. Cancer Lett 476: 1-12. https://doi.org/10.1016/j.canlet.2020.02.002

2. QT Ostrom, H Gittleman, P Farah, A Ondracek, Y Chen, Y Wolinsky, NE Stroup, C Kruchko, JS Barnholtz-Sloan (2013) CBTRUS statistical report: Primary brain and central nervous system tumors diagnosed in the United States in 2006-2010. Neuro Oncol 15 Suppl 2: ii1-56. https://doi.org/10.1093/neuonc/not151

3. QT Ostrom, G Cioffi, H Gittleman, N Patil, K Waite, C Kruchko, JS Barnholtz-Sloan (2019) CBTRUS Statistical Report: Primary Brain and Other Central Nervous System Tumors Diagnosed in the United States in 2012-2016. Neuro Oncol 21: v1-v100. https://doi.org/10.1093/neuonc/noz150

4. QT Ostrom, PM de Blank, C Kruchko et al (2015) Alex's Lemonade Stand Foundation Infant and Childhood Primary Brain and Central Nervous System Tumors Diagnosed in the United States in 2007-2011. Neuro Oncol 16 Suppl 10: x1-x36. https://doi.org/10.1093/neuonc/nou327

5. QT Ostrom, L Bauchet, FG Davis et al (2014) The epidemiology of glioma in adults: a "state of the science" review. Neuro Oncol 16: 896-913. https://doi.org/10.1093/neuonc/nou087

6. M Dobes, VG Khurana, B Shadbolt, S Jain, SF Smith, R Smee, M Dexter, R Cook (2011) Increasing incidence of glioblastoma multiforme and meningioma, and decreasing incidence of Schwannoma (2000-2008): Findings of a multicenter Australian study. Surg Neurol Int 2: 176. https://doi.org/10.4103/2152-7806.90696

7. D Gigineishvili, N Shengelia, G Shalashvili, S Rohrmann, A Tsiskaridze, R Shakarishvili (2013) Primary brain tumour epidemiology in Georgia: first-year results of a population-based study. J Neurooncol 112: 241-6. https://doi.org/10.1007/s11060-013-1054-1

8. P Bandopadhayay, G Bergthold, WB London et al (2014) Long-term outcome of 4,040 children diagnosed with pediatric low-grade gliomas: an analysis of the Surveillance Epidemiology and End Results (SEER) database. Pediatr Blood Cancer 61: 1173-9. https://doi.org/10.1002/pbc.24958

9. GT Armstrong, Q Liu, Y Yasui et al (2009) Long-term outcomes among adult survivors of childhood central nervous system malignancies in the Childhood Cancer Survivor Study. J Natl Cancer Inst 101: 946-58. https://doi.org/10.1093/jnci/djp148

10. JPM Pérez, J Muchart, VS López, MS Capella, N Salvador, SP Jaume, OC Martínez, AM La Madrid (2021) Targeted therapy for pediatric low-grade glioma. Childs Nerv Syst 37: 2511-20. https://doi.org/10.1007/s00381-021-05138-3

11. R Krishnatry, N Zhukova, AS Guerreiro Stucklin et al (2016) Clinical and treatment factors determining long-term outcomes for adult survivors of childhood low-grade glioma: A population-based study. Cancer 122: 1261-9. https://doi.org/10.1002/cncr.29907

12. H Davies, GR Bignell, C Cox et al (2002) Mutations of the BRAF gene in human cancer. Nature 417: 949-54. https://doi.org/10.1038/nature00766

13. S Ryall, M Zapotocky, K Fukuoka et al (2020) Integrated Molecular and Clinical Analysis of 1,000 Pediatric Low-Grade Gliomas. Cancer Cell 37: 569-83.e5. https://doi.org/10.1016/j.ccell.2020.03.011

14. PT Wan, MJ Garnett, SM Roe et al (2004) Mechanism of activation of the RAF-ERK signaling pathway by oncogenic mutations of B-RAF. Cell 116: 855-67. https://doi.org/10.1016/s0092-8674(04)00215-6

15. JY Tan, IVS Wijesinghe, MN Alfarizal Kamarudin, I Parhar (2021) Paediatric Gliomas: BRAF and Histone H3 as Biomarkers, Therapy and Perspective of Liquid Biopsies. Cancers (Basel) 13: https://doi.org/10.3390/cancers13040607

16. CL Penman, C Faulkner, SP Lowis, KM Kurian (2015) Current Understanding of BRAF Alterations in Diagnosis, Prognosis, and Therapeutic Targeting in Pediatric Low-Grade Gliomas. Front Oncol 5: 54. https://doi.org/10.3389/fonc.2015.00054

17. A Lassaletta, M Zapotocky, M Mistry et al (2017) Therapeutic and Prognostic Implications of BRAF V600E in Pediatric Low-Grade Gliomas. J Clin Oncol 35: 2934-41. https://doi.org/10.1200/jco.2016.71.8726

18. M Mistry, N Zhukova, D Merico et al (2015) BRAF mutation and CDKN2A deletion define a clinically distinct subgroup of childhood secondary high-grade glioma. J Clin Oncol 33: 1015-22. https://doi.org/10.1200/jco.2014.58.3922

19. MJ Lim-Fat, KW Song, JB Iorgulescu et al (2021) Clinical, radiological and genomic features and targeted therapy in BRAF V600E mutant adult glioblastoma. J Neurooncol 152: 515-22. https://doi.org/10.1007/s11060-021-03719-5

20. V Subbiah, RJ Kreitman, ZA Wainberg et al (2023) Dabrafenib plus trametinib in BRAFV600E-mutated rare cancers: the phase 2 ROAR trial. Nat Med 29: 1103-12. https://doi.org/10.1038/s41591-023-02321-8

21. PY Wen, A Stein, M van den Bent et al (2022) Dabrafenib plus trametinib in patients with BRAF(V600E)-mutant low-grade and high-grade glioma (ROAR): a multicentre, open-label, single-arm, phase 2, basket trial. Lancet Oncol 23: 53-64. https://doi.org/10.1016/s1470-2045(21)00578-7

22. T Kaley, M Touat, V Subbiah et al (2018) BRAF Inhibition in BRAF(V600)-Mutant Gliomas: Results From the VE-BASKET Study. J Clin Oncol 36: 3477-84. https://doi.org/10.1200/jco.2018.78.9990

23. KC Schreck, SA Grossman, CA Pratilas (2019) BRAF Mutations and the Utility of RAF and MEK Inhibitors in Primary Brain Tumors. Cancers (Basel) 11: https://doi.org/10.3390/cancers11091262

24. NF Brown, T Carter, N Kitchen, P Mulholland (2017) Dabrafenib and trametinib in BRAFV600E mutated glioma. CNS Oncol 6: 291-6. https://doi.org/10.2217/cns-2017-0006

25. MG Vander Heiden, LC Cantley, CB Thompson (2009) Understanding the Warburg effect: the metabolic requirements of cell proliferation. Science 324: 1029-33. https://doi.org/10.1126/science.1160809

26. AJ King, MR Arnone, MR Bleam et al (2013) Dabrafenib; preclinical characterization, increased efficacy when combined with trametinib, while BRAF/MEK tool combination reduced skin lesions. PLoS One 8: e67583. https://doi.org/10.1371/journal.pone.0067583

27. SA Luebker, SA Koepsell (2019) Diverse Mechanisms of BRAF Inhibitor Resistance in Melanoma Identified in Clinical and Preclinical Studies. Front Oncol 9: 268. https://doi.org/10.3389/fonc.2019.00268

28. C Sun, L Wang, S Huang et al (2014) Reversible and adaptive resistance to BRAF(V600E) inhibition in melanoma. Nature 508: 118-22. https://doi.org/10.1038/nature13121

29. T Rosenberg, KK Yeo, A Mauguen et al (2022) Upfront molecular targeted therapy for the treatment of BRAF-mutant pediatric high-grade glioma. Neuro Oncol 24: 1964-75. https://doi.org/10.1093/neuonc/noac096

30. MI Barbato, J Nashed, D Bradford et al (2023) FDA Approval Summary: Dabrafenib in combination with trametinib for BRAF V600E mutation-positive low-grade glioma. Clin Cancer Res: https://doi.org/10.1158/1078-0432.Ccr-23-1503

31. P Corrie, N Meyer, R Berardi, M Guidoboni, M Schlueter, S Kolovos, B Macabeo, JB Trouiller, P Laramée (2022) Comparative efficacy and safety of targeted therapies for BRAF-mutant unresectable or metastatic melanoma: Results from a systematic literature review and a network meta-analysis. Cancer Treat Rev 110: 102463. https://doi.org/10.1016/j.ctrv.2022.102463

32. J Li, M Sasane, J Zhao, VG Horton, P Zhang, ML Ricculli, ZY Zhou, J Signorovitch (2018) Comparative Efficacy of Treatments for Previously Treated Advanced or Metastatic Non-Small-Cell Lung Cancer: A Network Meta-Analysis. Adv Ther 35: 1035-48. https://doi.org/10.1007/s12325-018-0734-9

33. E Bouffet, B Geoerger, C Moertel et al (2023) Efficacy and Safety of Trametinib Monotherapy or in Combination With Dabrafenib in Pediatric BRAF V600-Mutant Low-Grade Glioma. J Clin Oncol 41: 664-74. https://doi.org/10.1200/jco.22.01000

34. MJ Page, JE McKenzie, PM Bossuyt et al (2021) The PRISMA 2020 statement: an updated guideline for reporting systematic reviews. Syst Rev 10: 89. https://doi.org/10.1186/s13643-021-01626-4

35. P Tsirogiannis, DR Reissmann, G Heydecke (2016) Evaluation of the marginal fit of single-unit, complete-coverage ceramic restorations fabricated after digital and conventional impressions: A systematic review and meta-analysis. J Prosthet Dent 116: 328-35.e2. https://doi.org/10.1016/j.prosdent.2016.01.028

36. J Davidenko, C Antzelevitch (1985) The effects of milrinone on action potential characteristics, conduction, automaticity, and reflected reentry in isolated myocardial fibers. J Cardiovasc Pharmacol 7: 341-9. https://doi.org/10.1097/00005344-198503000-00021

37. CB Begg, M Mazumdar (1994) Operating characteristics of a rank correlation test for publication bias. Biometrics 50: 1088-101.

38. M Egger, G Davey Smith, M Schneider, C Minder (1997) Bias in meta-analysis detected by a simple, graphical test. Bmj 315: 629-34. https://doi.org/10.1136/bmj.315.7109.629

39. JW Tsai, JJ Choi, H Ouaalam et al (2023) Integrated response analysis of pediatric low-grade gliomas during and after targeted therapy treatment. Neurooncol Adv 5: vdac182. https://doi.org/10.1093/noajnl/vdac182

40. DR Hargrave, K Terashima, J Hara et al (2023) Phase II Trial of Dabrafenib Plus Trametinib in Relapsed/Refractory BRAF V600-Mutant Pediatric High-Grade Glioma. J Clin Oncol 41: 5174-83. https://doi.org/10.1200/jco.23.00558

41. M Kushnirsky, LG Feun, SH Gultekin, MI de la Fuente (2020) Prolonged Complete Response With Combined Dabrafenib and Trametinib After BRAF Inhibitor Failure in BRAF-Mutant Glioblastoma. JCO Precis Oncol 4: https://doi.org/10.1200/po.19.00272

42. E Bouffet, JR Hansford, ML Garrè et al (2023) Dabrafenib plus Trametinib in Pediatric Glioma with BRAF V600 Mutations. N Engl J Med 389: 1108-20. https://doi.org/10.1056/NEJMoa2303815

43. M Geurts, MJ van den Bent (2019) On high-risk, low-grade glioma: What distinguishes high from low? Cancer 125: 174-6. https://doi.org/10.1002/cncr.31834

44. L Nobre, M Zapotocky, V Ramaswamy et al (2020) Outcomes of BRAF V600E Pediatric Gliomas Treated With Targeted BRAF Inhibition. JCO Precis Oncol 4: https://doi.org/10.1200/po.19.00298

45. AS Wen P, Yung-Jue B et al (2018) Efcacy and safety of dabrafenib + trametinib in patients with recurrent/ refractory braf v600e–mutated high-grade gliomA (HGG). Neuro Oncol 20:238.: https://doi.org/https://doi.org/10.1093/neuonc/noy148.986

46. EY Chu, KA Wanat, CJ Miller et al (2012) Diverse cutaneous side effects associated with BRAF inhibitor therapy: a clinicopathologic study. J Am Acad Dermatol 67: 1265-72. https://doi.org/10.1016/j.jaad.2012.04.008

47. L Peng, Y Wang, Y Hong, X Ye, P Shi, J Zhang, Q Zhao (2017) Incidence and relative risk of cutaneous squamous cell carcinoma with single-agent BRAF inhibitor and dual BRAF/MEK inhibitors in cancer patients: a meta-analysis. Oncotarget 8: 83280-91. https://doi.org/10.18632/oncotarget.21059

48. A Hauschild, JJ Grob, LV Demidov et al (2012) Dabrafenib in BRAF-mutated metastatic melanoma: a multicentre, open-label, phase 3 randomised controlled trial. Lancet 380: 358-65. https://doi.org/10.1016/s0140-6736(12)60868-x

49. AM Menzies, GV Long (2014) Dabrafenib and trametinib, alone and in combination for BRAF-mutant metastatic melanoma. Clin Cancer Res 20: 2035-43. https://doi.org/10.1158/1078-0432.Ccr-13-2054

50. SJ Welsh, PG Corrie (2015) Management of BRAF and MEK inhibitor toxicities in patients with metastatic melanoma. Ther Adv Med Oncol 7: 122-36. https://doi.org/10.1177/1758834014566428

51. CY Ho, BC Mobley, H Gordish-Dressman et al (2015) A clinicopathologic study of diencephalic pediatric low-grade gliomas with BRAF V600 mutation. Acta Neuropathol 130: 575-85. https://doi.org/10.1007/s00401-015-1467-3

52. DR Hargrave, E Bouffet, U Tabori et al (2019) Efficacy and Safety of Dabrafenib in Pediatric Patients with BRAF V600 Mutation-Positive Relapsed or Refractory Low-Grade Glioma: Results from a Phase I/IIa Study. Clin Cancer Res 25: 7303-11. https://doi.org/10.1158/1078-0432.Ccr-19-2177

53. T Nicolaides, KJ Nazemi, J Crawford et al (2020) Phase I study of vemurafenib in children with recurrent or progressive BRAF(V600E) mutant brain tumors: Pacific Pediatric Neuro-Oncology Consortium study (PNOC-002). Oncotarget 11: 1942-52. https://doi.org/10.18632/oncotarget.27600

Figure Legends

**Figure 1.**PRISMA flow chart for study screening and inclusion.

**Figure 2.** Forest plot for PFS (a) and OS (b).

**Figure 3.** Forest plot for AEs (a) and death events (b).

**Supplementary material file**

**Supplementary Table 1.**Search strategy.

**Supplementary Table 2.**Publication bias and heterogeneity of summarized outcomes.

**Supplementary Figure 1.**Forest plot for PFS rate (a) and Subgroups analysis of PFS rate by the intervention (b).

**Supplementary Figure 2.** Forest plot for PR (a) and CR (b).

**Supplementary Figure 3.**Forest plot for ORR (a) and RR (b).

**Supplementary Figure 4.** Subgroups analysis of RR by age (a) and by the intervention (b).

**Supplementary Figure 5.** Subgroups analysis of AEs by age (a) and by the intervention (b).

**Supplementary Figure 6.**The funnel plot for PFS

**Supplementary Figure 7.**The funnel plot for PFS rate

**Supplementary Figure 8.**The funnel plot for OS

**Supplementary Figure 9.**The funnel plot for PR

**Supplementary Figure 10.**The funnel plot for CR

**Supplementary Figure 11.**The funnel plot for ORR

**Supplementary Figure 12.**The funnel plot for RR

**Supplementary Figure 13.**The funnel plot for AEs

**Supplementary Figure 14.**The funnel plot for death events

**Supplementary Figure 15.**The funnel plot for PFS

**Supplementary Figure 16.**The funnel plot for PFS rate

**Supplementary Figure 17.**The funnel plot for OS

**Supplementary Figure 18.**The funnel plot for PR

**Supplementary Figure 19.**The funnel plot for CR

**Supplementary Figure 20.**The funnel plot for ORR

**Supplementary Figure 21.**Sensitivity analysis of RR

**Supplementary Figure 22.**Sensitivity analysis of AEs

**Supplementary Figure 23.**Sensitivity analysis of death events

**Table 1.** Baseline characteristics of 8 included studies.

| Study ID | Country | Simple size | Study design | Clinical diagnosis | Age (years old) | Gender, female, n (%) | Intervention (the experimentalgroup) | Intervention (the control group) | Outcomes |
| --- | --- | --- | --- | --- | --- | --- | --- | --- | --- |
| Wen 2022 | USA | 58 | cohort study (single-arm) | BRAFV600E  HGGs and LGGs | HGGs: 42 (18−72)  LGGs: 33 (18−58) | 53.45 | oral dabrafenib (150 mg orally twice daily) and trametinib (2 mg orally once daily) | NA | PFS, OS, RR, AEs, death events |
| Hargrave 2023 | England | 41 | cohort study (single-arm) | BRAFV600E PHGG | 13 (2.0-17.0) | 56.1 | Oral dabrafenib (5.25 mg/kg/d for patients younger than 12 years; 4.5 mg/kg/d for patients age 12 years and older) and trametinib (0.032mg/kg/d for patients younger than 6 years; 0.025 mg/kg/d for patients age 6 years and older) | NA | PFS, PSF rate, ORR, CR, PR, AEs, death events |
| LimFat 2021 | Canada | 5 | retrospective study (single-arm) | BRAF v600-mutant glioblastoma | 41 (22–69) | NA | oral dabrafenib (150 mg twice daily) and trametinib (2mg once daily) | NA | PFS, OS, RR |
| Tsai 2022 | USA | 20 | retrospective study (single-arm) | BRAF v600-mutant LGG | 4.42 (0.13–25.84) | NA | oral dabrafenib or trametinib | NA | PSF rate, RR |
| Perez 2021 | Spain | 23 | retrospective study (single-arm) | BRAF v600-mutant PLGG | 3.2 ( 0.4–17.8) | 47.83 | oral dabrafenib or trametinib | NA | PSF rate, ORR, RR |
| Subbiah 2023 | USA | 58 | cohort study (single-arm) | BRAFV600E HGGs and LGGs | HGGs: 41.9 (sd=14.70)  LGGs: 33.1 (sd=11.51) | 53.45 | oral dabrafenib (150 mg twice daily) and oral trametinib (2mg once daily) | NA | CR, PR, AEs |
| Bouffet 2022 | Canada | 49 (E/C:36/13) | cohort study (double-arm) | BRAF v600-mutant PLGG | 9.2 | 51.02 | Dabrafenib plus trametinib combination therapy | Trametinib monotherapy | RR, AEs |
| Rosenberg 2022 | USA | 14 (E/C:11/3) | retrospective study (double-arm) | BRAF v600-mutant PHGG | 11.29 | NA | Dabrafenib plus trametinib combination therapy | Dabrafenib monotherapy | AEs |

**Abbreviations:** NA:not available, E/C: the experimental group:the control group, PFS:progression free survival, OS:overall survival, PR: partial response, CR:complete response, ORR:objective response rate, RR:response rate, AEs: adverse events, HGG: high-grade glioma, LGG: low-grade glioma, PHGG: pediatric high-grade gliomas, PLGG: pediatric low-grade glioma.

**Table 2. Quality assessment of included studies by MINORS**

| Study | 1 | 2 | 3 | 4 | 5 | 6 | 7 | 8 | 9 | 10 | 11 | 12 | Total |
| --- | --- | --- | --- | --- | --- | --- | --- | --- | --- | --- | --- | --- | --- |
| Wen 2022 | 2 | 2 | 2 | 2 | 1 | 2 | 1 | 2 |  |  |  |  | 14 |
| Hargrave 2023 | 2 | 2 | 2 | 2 | 2 | 2 | 2 | 2 |  |  |  |  | 16 |
| Lim‑Fat 2021 | 2 | 2 | 2 | 2 | 2 | 2 | 1 | 1 |  |  |  |  | 14 |
| Tsai 2022 | 2 | 2 | 2 | 2 | 1 | 2 | 2 | 2 |  |  |  |  | 15 |
| Perez 2021 | 2 | 2 | 2 | 2 | 2 | 2 | 2 | 2 |  |  |  |  | 16 |
| Subbiah 2023 | 2 | 2 | 2 | 2 | 1 | 2 | 2 | 2 |  |  |  |  | 15 |
| Bouffet 2022 | 2 | 2 | 2 | 2 | 2 | 2 | 2 | 2 | 2 | 2 | 2 | 2 | 24 |
| Rosenberg 2022 | 2 | 2 | 2 | 2 | 2 | 2 | 1 | 1 | 1 | 2 | 1 | 2 | 20 |

1.A stated aim of the study; 2.Inclusion of consecutive patients; 3.Prospective collection of data; 4.Endpoint appropriate to the study aim; 5.Unbiased evaluation of endpoints; 6.Follow-up period appropriate to the major endpoint; 7.Loss to follow up not exceeding 5%; 8.Prospective calculation of the sample size; 9.A control group having the gold standard intervention; 10.Contemporary groups; 11.Baseline equivalence of groups; 12.Statistical analyses adapted to the study design;
