# Supplementary figures and images for: The effects of dabrafenib and/or trametinib treatment in Braf V600-mutant glioma: a systematic review and meta-analysis
Source: Neurosurg Rev. 2024 Aug 22;47(1):458. doi: 10.1007/s10143-024-02664-x (PMC11341626; doi:10.1007/s10143-024-02664-x)

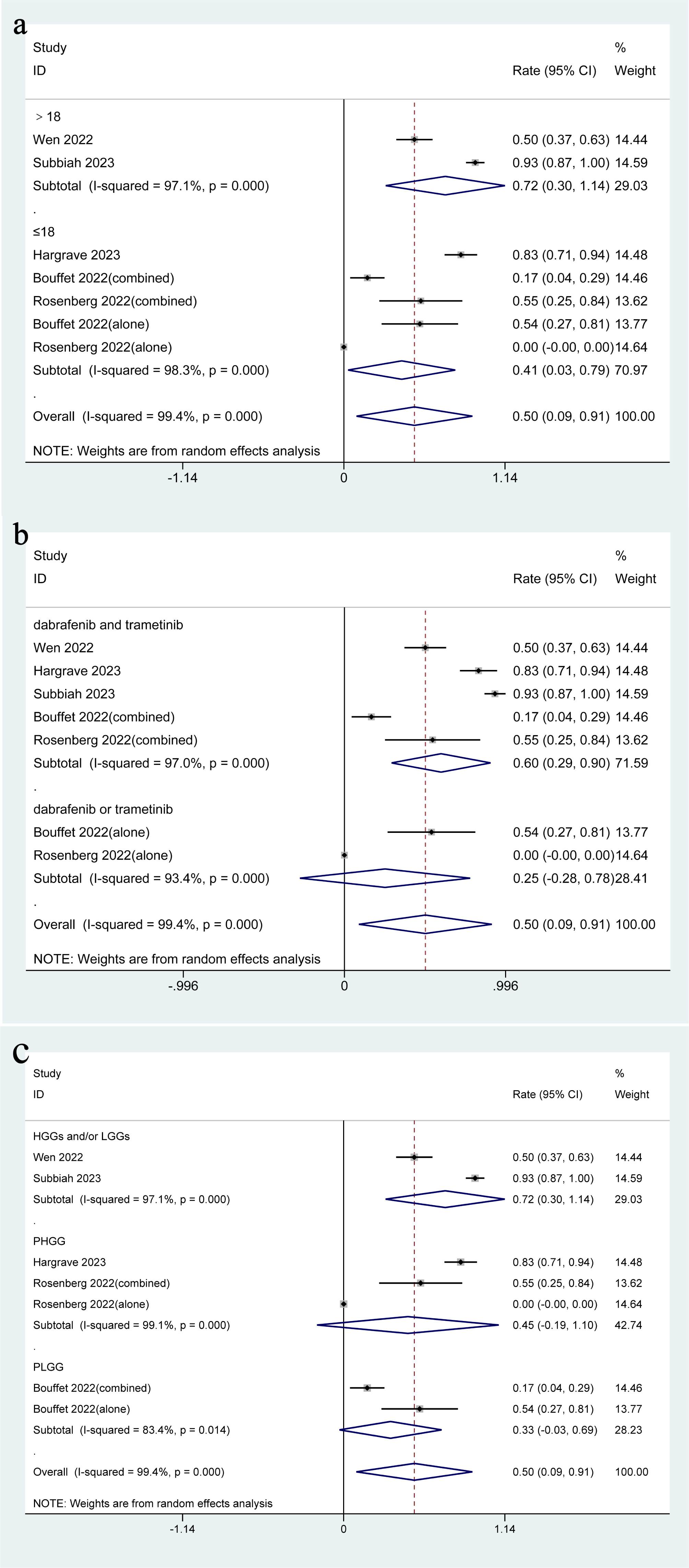

Supplement: Supplementary file 1 — Supplementary file1 Supplementary Figure 1.Forest plot for PFS rate (a) and Subgroups analysis of PFS rate by the intervention (b). Supplementary Figure 2. Forest plot for PR (a) and CR (b). Supplementary Figure 3.Forest plot for ORR (a) and RR (b). Supplementary Figure 4. Subgroups analysis of RR by age (a) and by the intervention (b). Supplementary Figure 5. Subgroups analysis of AEs by age (a) and by the intervention (b). Supplementary Figure 6.The funnel plot for PFS. Supplementary Figure 7.The funnel plot for PFS rate. Supplementary Figure 8.The funnel plot for OS. Supplementary Figure 9.The funnel plot for PR. Supplementary Figure 10.The funnel plot for CR Supplementary Figure 11.The funnel plot for ORR. Supplementary Figure 12.The funnel plot for RR. Supplementary Figure 13.The funnel plot for AEs Supplementary Figure 14.The funnel plot for death events. Supplementary Figure 15.The funnel plot for PFS. Supplementary Figure 16.The funnel plot for PFS rate. Supplementary Figure 17.The funnel plot for OS Supplementary Figure 18.The funnel plot for PR. Supplementary Figure 19.The funnel plot for CR. Supplementary Figure 20.The funnel plot for ORR. Supplementary Figure 21.Sensitivity analysis of RR. Supplementary Figure 22.Sensitivity analysis of AEs. Supplementary Figure 23.Sensitivity analysis of death events (ZIP 1916 KB) [file 10143_2024_2664_MOESM1_ESM.zip › Supplementary Figure 1.jpg]

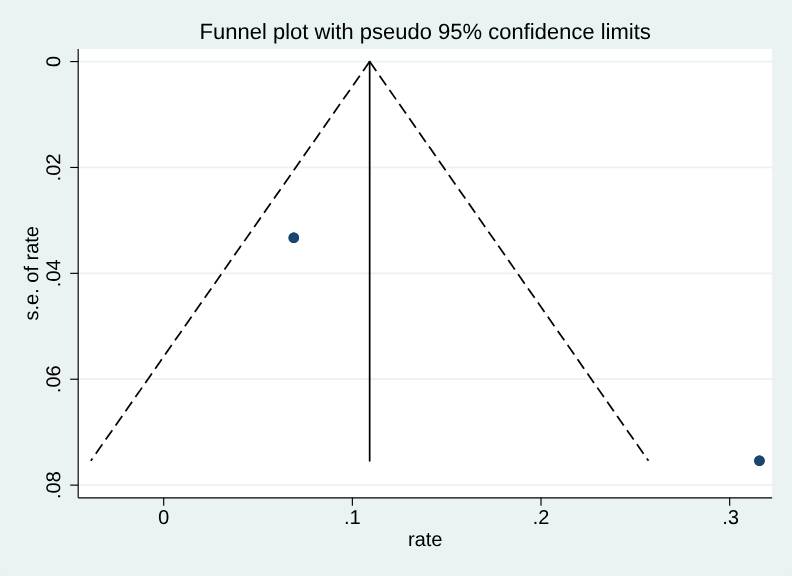

Supplement: Supplementary file 1 — Supplementary file1 Supplementary Figure 1.Forest plot for PFS rate (a) and Subgroups analysis of PFS rate by the intervention (b). Supplementary Figure 2. Forest plot for PR (a) and CR (b). Supplementary Figure 3.Forest plot for ORR (a) and RR (b). Supplementary Figure 4. Subgroups analysis of RR by age (a) and by the intervention (b). Supplementary Figure 5. Subgroups analysis of AEs by age (a) and by the intervention (b). Supplementary Figure 6.The funnel plot for PFS. Supplementary Figure 7.The funnel plot for PFS rate. Supplementary Figure 8.The funnel plot for OS. Supplementary Figure 9.The funnel plot for PR. Supplementary Figure 10.The funnel plot for CR Supplementary Figure 11.The funnel plot for ORR. Supplementary Figure 12.The funnel plot for RR. Supplementary Figure 13.The funnel plot for AEs Supplementary Figure 14.The funnel plot for death events. Supplementary Figure 15.The funnel plot for PFS. Supplementary Figure 16.The funnel plot for PFS rate. Supplementary Figure 17.The funnel plot for OS Supplementary Figure 18.The funnel plot for PR. Supplementary Figure 19.The funnel plot for CR. Supplementary Figure 20.The funnel plot for ORR. Supplementary Figure 21.Sensitivity analysis of RR. Supplementary Figure 22.Sensitivity analysis of AEs. Supplementary Figure 23.Sensitivity analysis of death events (ZIP 1916 KB) [file 10143_2024_2664_MOESM1_ESM.zip › Supplementary Figure 10.jpg]

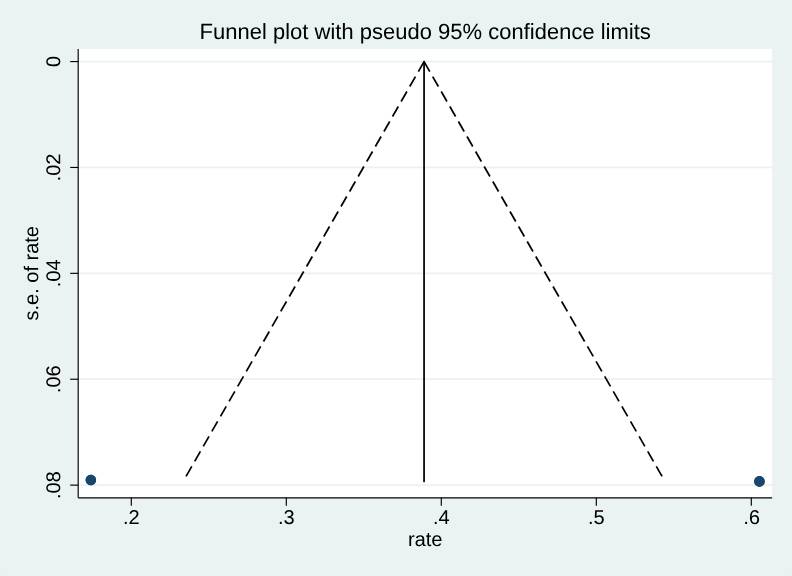

Supplement: Supplementary file 1 — Supplementary file1 Supplementary Figure 1.Forest plot for PFS rate (a) and Subgroups analysis of PFS rate by the intervention (b). Supplementary Figure 2. Forest plot for PR (a) and CR (b). Supplementary Figure 3.Forest plot for ORR (a) and RR (b). Supplementary Figure 4. Subgroups analysis of RR by age (a) and by the intervention (b). Supplementary Figure 5. Subgroups analysis of AEs by age (a) and by the intervention (b). Supplementary Figure 6.The funnel plot for PFS. Supplementary Figure 7.The funnel plot for PFS rate. Supplementary Figure 8.The funnel plot for OS. Supplementary Figure 9.The funnel plot for PR. Supplementary Figure 10.The funnel plot for CR Supplementary Figure 11.The funnel plot for ORR. Supplementary Figure 12.The funnel plot for RR. Supplementary Figure 13.The funnel plot for AEs Supplementary Figure 14.The funnel plot for death events. Supplementary Figure 15.The funnel plot for PFS. Supplementary Figure 16.The funnel plot for PFS rate. Supplementary Figure 17.The funnel plot for OS Supplementary Figure 18.The funnel plot for PR. Supplementary Figure 19.The funnel plot for CR. Supplementary Figure 20.The funnel plot for ORR. Supplementary Figure 21.Sensitivity analysis of RR. Supplementary Figure 22.Sensitivity analysis of AEs. Supplementary Figure 23.Sensitivity analysis of death events (ZIP 1916 KB) [file 10143_2024_2664_MOESM1_ESM.zip › Supplementary Figure 11.jpg]

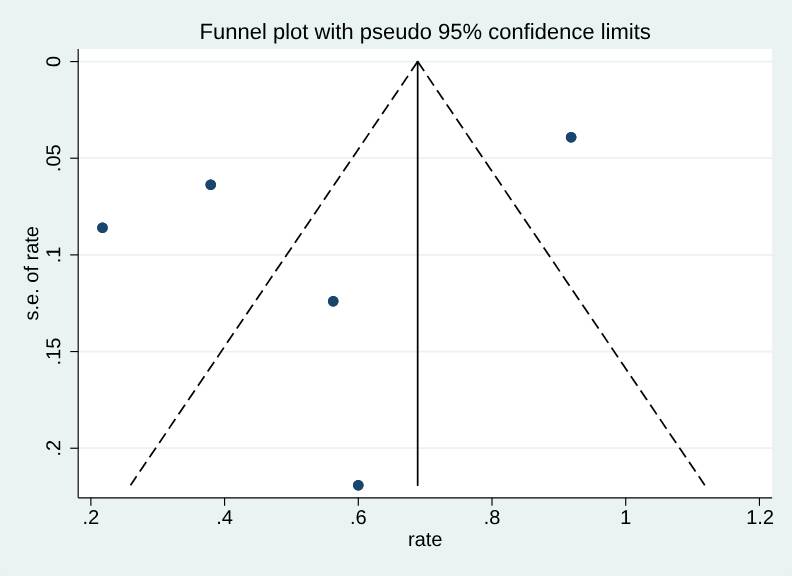

Supplement: Supplementary file 1 — Supplementary file1 Supplementary Figure 1.Forest plot for PFS rate (a) and Subgroups analysis of PFS rate by the intervention (b). Supplementary Figure 2. Forest plot for PR (a) and CR (b). Supplementary Figure 3.Forest plot for ORR (a) and RR (b). Supplementary Figure 4. Subgroups analysis of RR by age (a) and by the intervention (b). Supplementary Figure 5. Subgroups analysis of AEs by age (a) and by the intervention (b). Supplementary Figure 6.The funnel plot for PFS. Supplementary Figure 7.The funnel plot for PFS rate. Supplementary Figure 8.The funnel plot for OS. Supplementary Figure 9.The funnel plot for PR. Supplementary Figure 10.The funnel plot for CR Supplementary Figure 11.The funnel plot for ORR. Supplementary Figure 12.The funnel plot for RR. Supplementary Figure 13.The funnel plot for AEs Supplementary Figure 14.The funnel plot for death events. Supplementary Figure 15.The funnel plot for PFS. Supplementary Figure 16.The funnel plot for PFS rate. Supplementary Figure 17.The funnel plot for OS Supplementary Figure 18.The funnel plot for PR. Supplementary Figure 19.The funnel plot for CR. Supplementary Figure 20.The funnel plot for ORR. Supplementary Figure 21.Sensitivity analysis of RR. Supplementary Figure 22.Sensitivity analysis of AEs. Supplementary Figure 23.Sensitivity analysis of death events (ZIP 1916 KB) [file 10143_2024_2664_MOESM1_ESM.zip › Supplementary Figure 12.jpg]

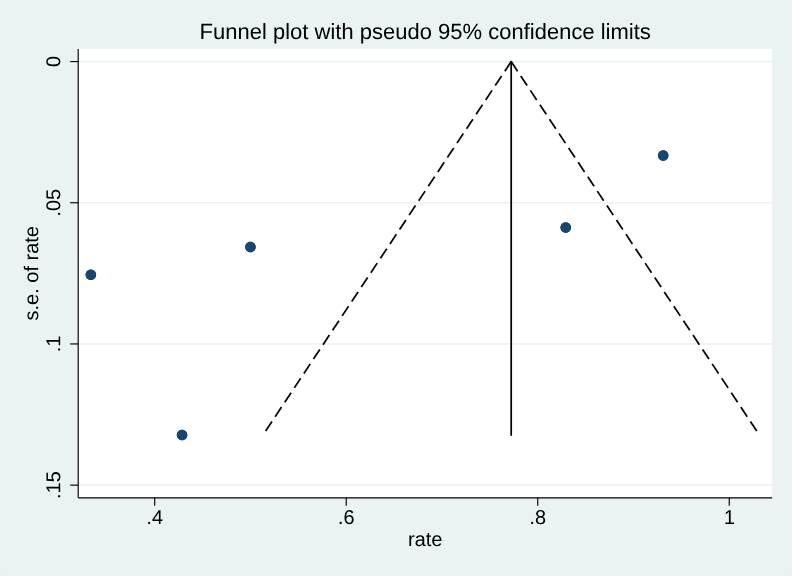

Supplement: Supplementary file 1 — Supplementary file1 Supplementary Figure 1.Forest plot for PFS rate (a) and Subgroups analysis of PFS rate by the intervention (b). Supplementary Figure 2. Forest plot for PR (a) and CR (b). Supplementary Figure 3.Forest plot for ORR (a) and RR (b). Supplementary Figure 4. Subgroups analysis of RR by age (a) and by the intervention (b). Supplementary Figure 5. Subgroups analysis of AEs by age (a) and by the intervention (b). Supplementary Figure 6.The funnel plot for PFS. Supplementary Figure 7.The funnel plot for PFS rate. Supplementary Figure 8.The funnel plot for OS. Supplementary Figure 9.The funnel plot for PR. Supplementary Figure 10.The funnel plot for CR Supplementary Figure 11.The funnel plot for ORR. Supplementary Figure 12.The funnel plot for RR. Supplementary Figure 13.The funnel plot for AEs Supplementary Figure 14.The funnel plot for death events. Supplementary Figure 15.The funnel plot for PFS. Supplementary Figure 16.The funnel plot for PFS rate. Supplementary Figure 17.The funnel plot for OS Supplementary Figure 18.The funnel plot for PR. Supplementary Figure 19.The funnel plot for CR. Supplementary Figure 20.The funnel plot for ORR. Supplementary Figure 21.Sensitivity analysis of RR. Supplementary Figure 22.Sensitivity analysis of AEs. Supplementary Figure 23.Sensitivity analysis of death events (ZIP 1916 KB) [file 10143_2024_2664_MOESM1_ESM.zip › Supplementary Figure 13.jpg]

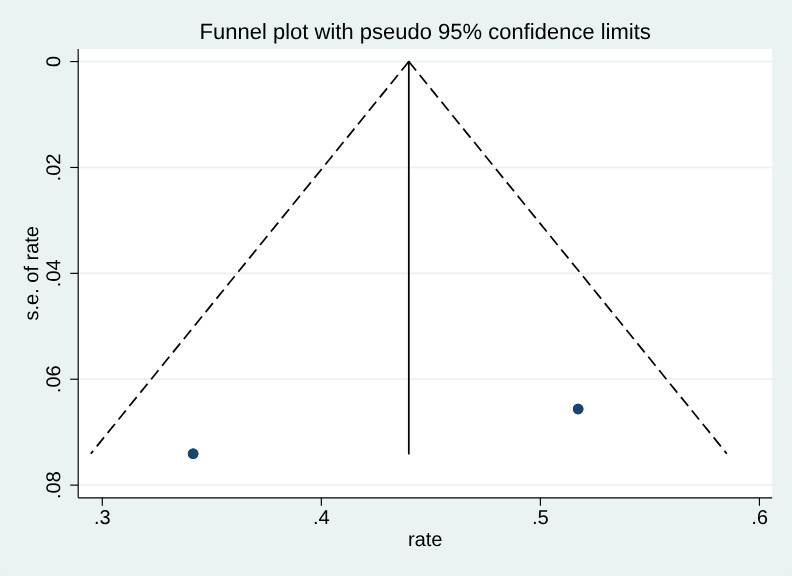

Supplement: Supplementary file 1 — Supplementary file1 Supplementary Figure 1.Forest plot for PFS rate (a) and Subgroups analysis of PFS rate by the intervention (b). Supplementary Figure 2. Forest plot for PR (a) and CR (b). Supplementary Figure 3.Forest plot for ORR (a) and RR (b). Supplementary Figure 4. Subgroups analysis of RR by age (a) and by the intervention (b). Supplementary Figure 5. Subgroups analysis of AEs by age (a) and by the intervention (b). Supplementary Figure 6.The funnel plot for PFS. Supplementary Figure 7.The funnel plot for PFS rate. Supplementary Figure 8.The funnel plot for OS. Supplementary Figure 9.The funnel plot for PR. Supplementary Figure 10.The funnel plot for CR Supplementary Figure 11.The funnel plot for ORR. Supplementary Figure 12.The funnel plot for RR. Supplementary Figure 13.The funnel plot for AEs Supplementary Figure 14.The funnel plot for death events. Supplementary Figure 15.The funnel plot for PFS. Supplementary Figure 16.The funnel plot for PFS rate. Supplementary Figure 17.The funnel plot for OS Supplementary Figure 18.The funnel plot for PR. Supplementary Figure 19.The funnel plot for CR. Supplementary Figure 20.The funnel plot for ORR. Supplementary Figure 21.Sensitivity analysis of RR. Supplementary Figure 22.Sensitivity analysis of AEs. Supplementary Figure 23.Sensitivity analysis of death events (ZIP 1916 KB) [file 10143_2024_2664_MOESM1_ESM.zip › Supplementary Figure 14.jpg]

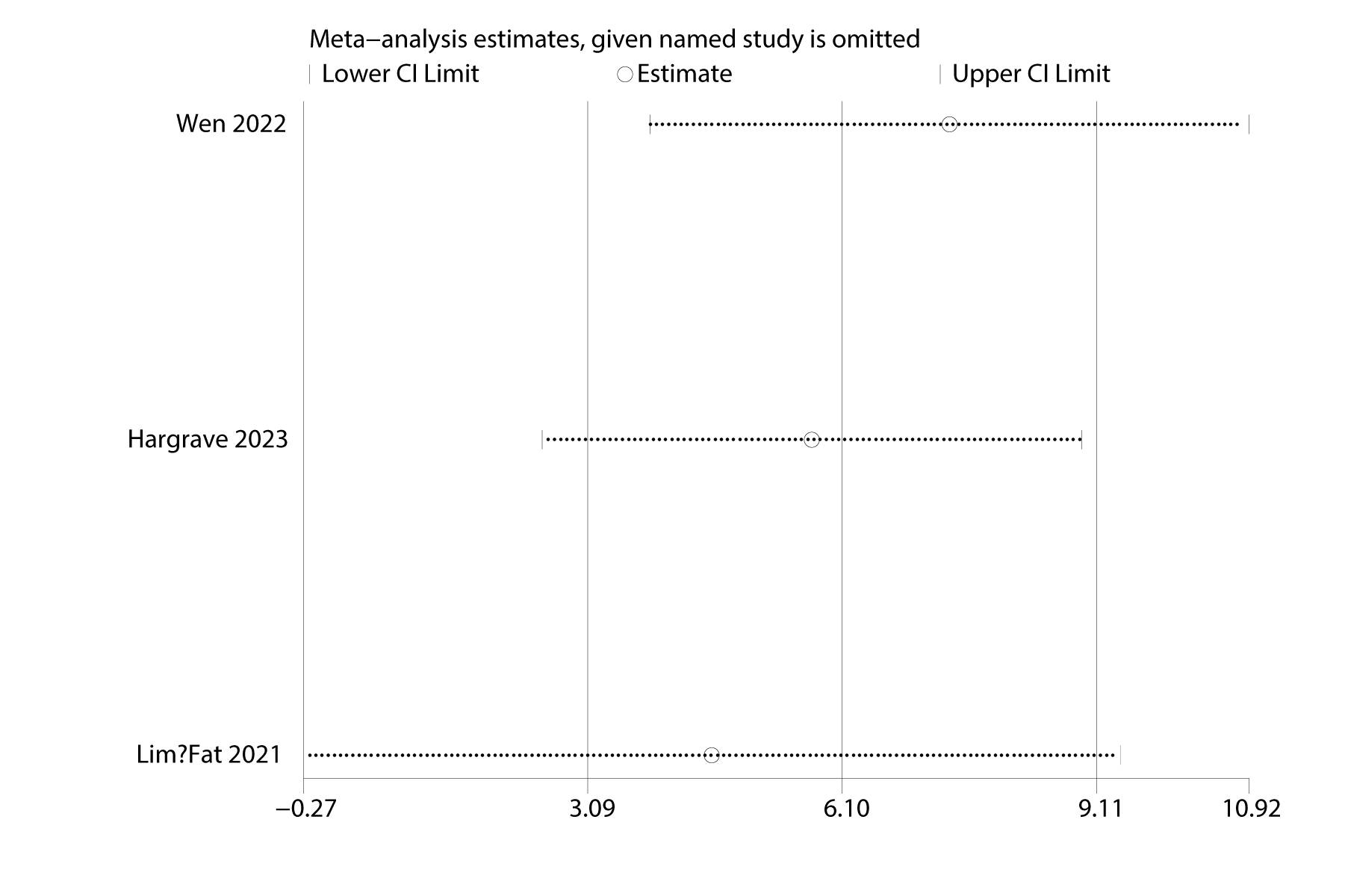

Supplement: Supplementary file 1 — Supplementary file1 Supplementary Figure 1.Forest plot for PFS rate (a) and Subgroups analysis of PFS rate by the intervention (b). Supplementary Figure 2. Forest plot for PR (a) and CR (b). Supplementary Figure 3.Forest plot for ORR (a) and RR (b). Supplementary Figure 4. Subgroups analysis of RR by age (a) and by the intervention (b). Supplementary Figure 5. Subgroups analysis of AEs by age (a) and by the intervention (b). Supplementary Figure 6.The funnel plot for PFS. Supplementary Figure 7.The funnel plot for PFS rate. Supplementary Figure 8.The funnel plot for OS. Supplementary Figure 9.The funnel plot for PR. Supplementary Figure 10.The funnel plot for CR Supplementary Figure 11.The funnel plot for ORR. Supplementary Figure 12.The funnel plot for RR. Supplementary Figure 13.The funnel plot for AEs Supplementary Figure 14.The funnel plot for death events. Supplementary Figure 15.The funnel plot for PFS. Supplementary Figure 16.The funnel plot for PFS rate. Supplementary Figure 17.The funnel plot for OS Supplementary Figure 18.The funnel plot for PR. Supplementary Figure 19.The funnel plot for CR. Supplementary Figure 20.The funnel plot for ORR. Supplementary Figure 21.Sensitivity analysis of RR. Supplementary Figure 22.Sensitivity analysis of AEs. Supplementary Figure 23.Sensitivity analysis of death events (ZIP 1916 KB) [file 10143_2024_2664_MOESM1_ESM.zip › Supplementary Figure 15.jpg]

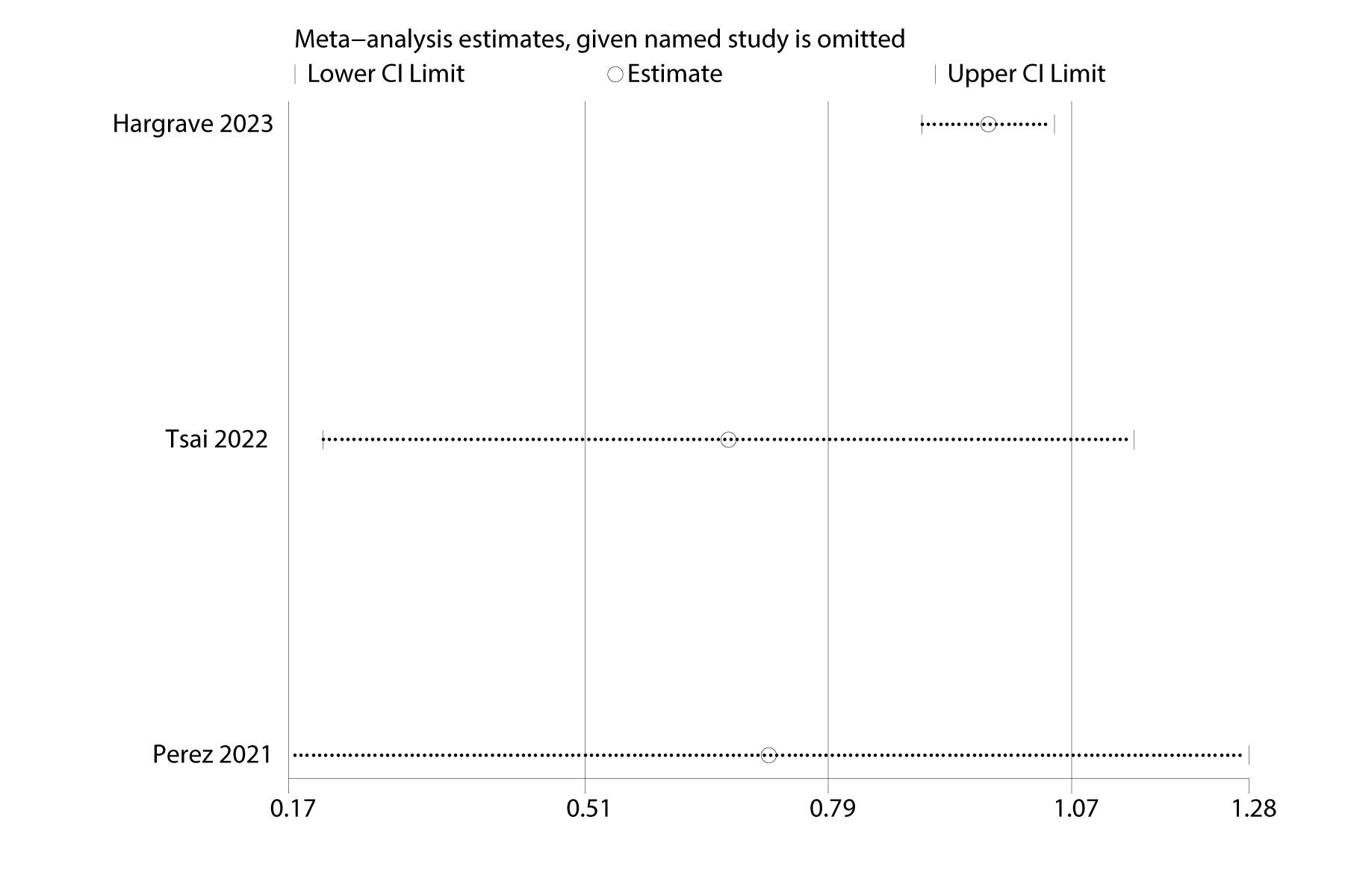

Supplement: Supplementary file 1 — Supplementary file1 Supplementary Figure 1.Forest plot for PFS rate (a) and Subgroups analysis of PFS rate by the intervention (b). Supplementary Figure 2. Forest plot for PR (a) and CR (b). Supplementary Figure 3.Forest plot for ORR (a) and RR (b). Supplementary Figure 4. Subgroups analysis of RR by age (a) and by the intervention (b). Supplementary Figure 5. Subgroups analysis of AEs by age (a) and by the intervention (b). Supplementary Figure 6.The funnel plot for PFS. Supplementary Figure 7.The funnel plot for PFS rate. Supplementary Figure 8.The funnel plot for OS. Supplementary Figure 9.The funnel plot for PR. Supplementary Figure 10.The funnel plot for CR Supplementary Figure 11.The funnel plot for ORR. Supplementary Figure 12.The funnel plot for RR. Supplementary Figure 13.The funnel plot for AEs Supplementary Figure 14.The funnel plot for death events. Supplementary Figure 15.The funnel plot for PFS. Supplementary Figure 16.The funnel plot for PFS rate. Supplementary Figure 17.The funnel plot for OS Supplementary Figure 18.The funnel plot for PR. Supplementary Figure 19.The funnel plot for CR. Supplementary Figure 20.The funnel plot for ORR. Supplementary Figure 21.Sensitivity analysis of RR. Supplementary Figure 22.Sensitivity analysis of AEs. Supplementary Figure 23.Sensitivity analysis of death events (ZIP 1916 KB) [file 10143_2024_2664_MOESM1_ESM.zip › Supplementary Figure 16.jpg]

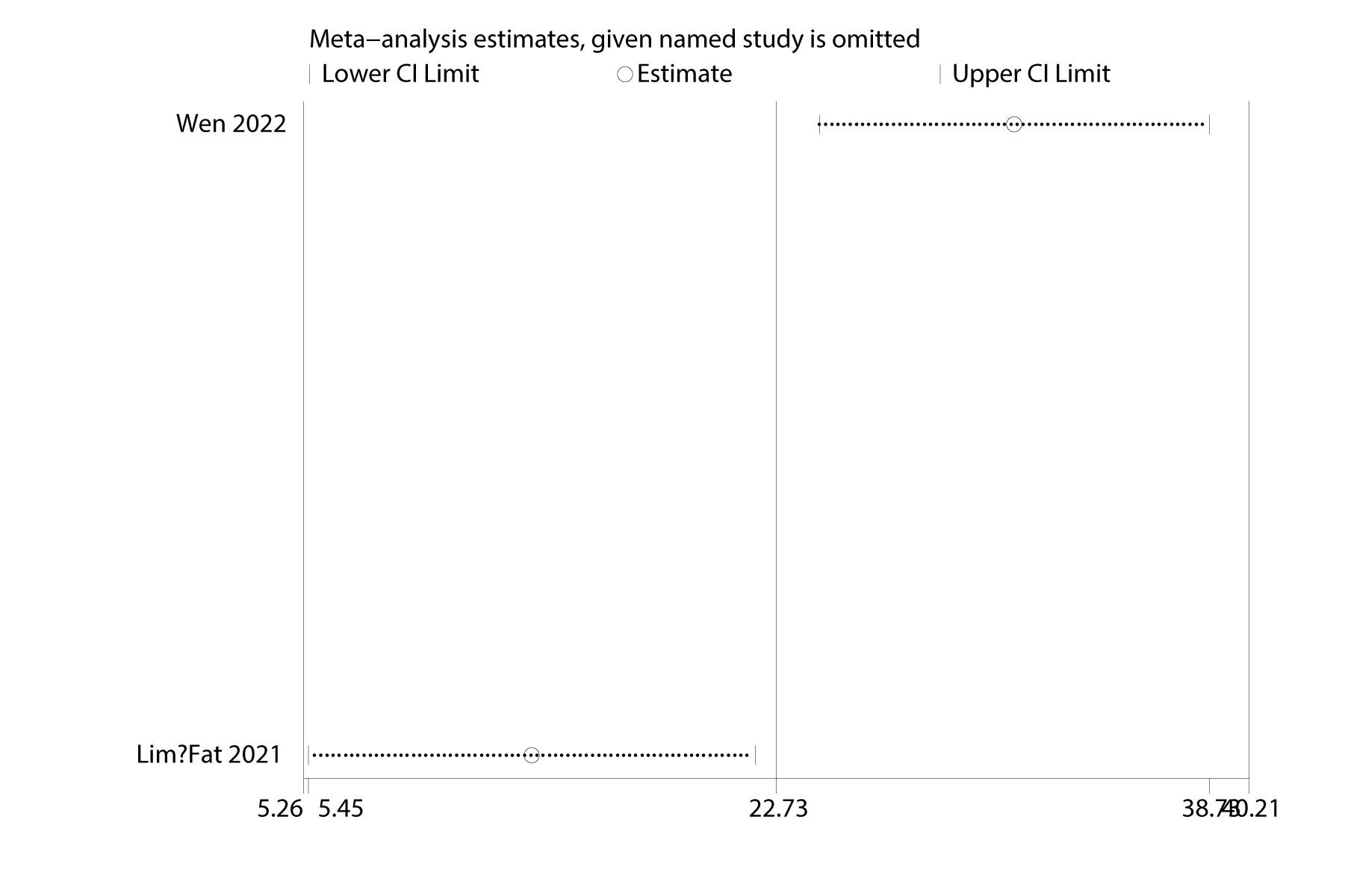

Supplement: Supplementary file 1 — Supplementary file1 Supplementary Figure 1.Forest plot for PFS rate (a) and Subgroups analysis of PFS rate by the intervention (b). Supplementary Figure 2. Forest plot for PR (a) and CR (b). Supplementary Figure 3.Forest plot for ORR (a) and RR (b). Supplementary Figure 4. Subgroups analysis of RR by age (a) and by the intervention (b). Supplementary Figure 5. Subgroups analysis of AEs by age (a) and by the intervention (b). Supplementary Figure 6.The funnel plot for PFS. Supplementary Figure 7.The funnel plot for PFS rate. Supplementary Figure 8.The funnel plot for OS. Supplementary Figure 9.The funnel plot for PR. Supplementary Figure 10.The funnel plot for CR Supplementary Figure 11.The funnel plot for ORR. Supplementary Figure 12.The funnel plot for RR. Supplementary Figure 13.The funnel plot for AEs Supplementary Figure 14.The funnel plot for death events. Supplementary Figure 15.The funnel plot for PFS. Supplementary Figure 16.The funnel plot for PFS rate. Supplementary Figure 17.The funnel plot for OS Supplementary Figure 18.The funnel plot for PR. Supplementary Figure 19.The funnel plot for CR. Supplementary Figure 20.The funnel plot for ORR. Supplementary Figure 21.Sensitivity analysis of RR. Supplementary Figure 22.Sensitivity analysis of AEs. Supplementary Figure 23.Sensitivity analysis of death events (ZIP 1916 KB) [file 10143_2024_2664_MOESM1_ESM.zip › Supplementary Figure 17.jpg]

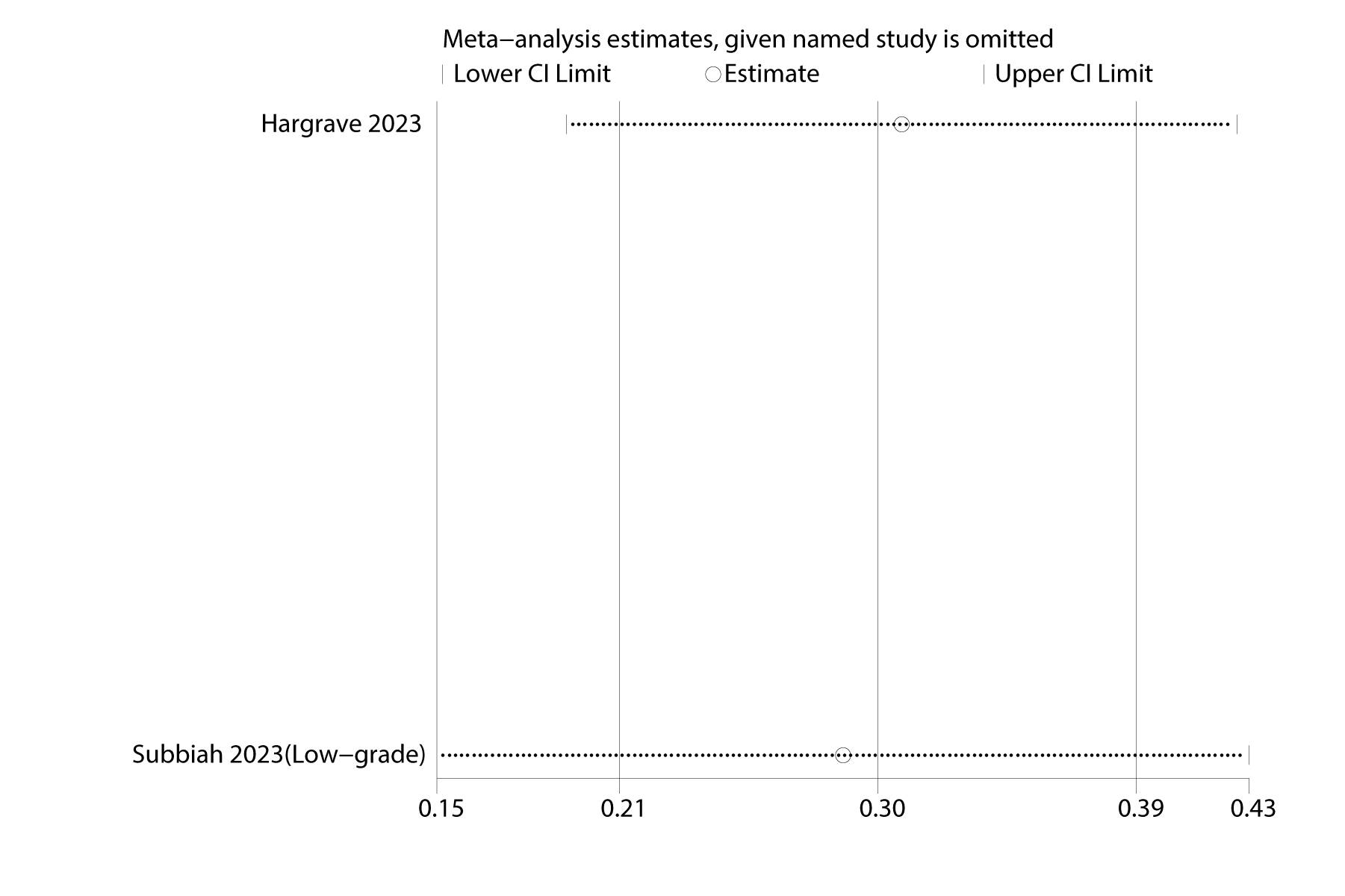

Supplement: Supplementary file 1 — Supplementary file1 Supplementary Figure 1.Forest plot for PFS rate (a) and Subgroups analysis of PFS rate by the intervention (b). Supplementary Figure 2. Forest plot for PR (a) and CR (b). Supplementary Figure 3.Forest plot for ORR (a) and RR (b). Supplementary Figure 4. Subgroups analysis of RR by age (a) and by the intervention (b). Supplementary Figure 5. Subgroups analysis of AEs by age (a) and by the intervention (b). Supplementary Figure 6.The funnel plot for PFS. Supplementary Figure 7.The funnel plot for PFS rate. Supplementary Figure 8.The funnel plot for OS. Supplementary Figure 9.The funnel plot for PR. Supplementary Figure 10.The funnel plot for CR Supplementary Figure 11.The funnel plot for ORR. Supplementary Figure 12.The funnel plot for RR. Supplementary Figure 13.The funnel plot for AEs Supplementary Figure 14.The funnel plot for death events. Supplementary Figure 15.The funnel plot for PFS. Supplementary Figure 16.The funnel plot for PFS rate. Supplementary Figure 17.The funnel plot for OS Supplementary Figure 18.The funnel plot for PR. Supplementary Figure 19.The funnel plot for CR. Supplementary Figure 20.The funnel plot for ORR. Supplementary Figure 21.Sensitivity analysis of RR. Supplementary Figure 22.Sensitivity analysis of AEs. Supplementary Figure 23.Sensitivity analysis of death events (ZIP 1916 KB) [file 10143_2024_2664_MOESM1_ESM.zip › Supplementary Figure 18.jpg]

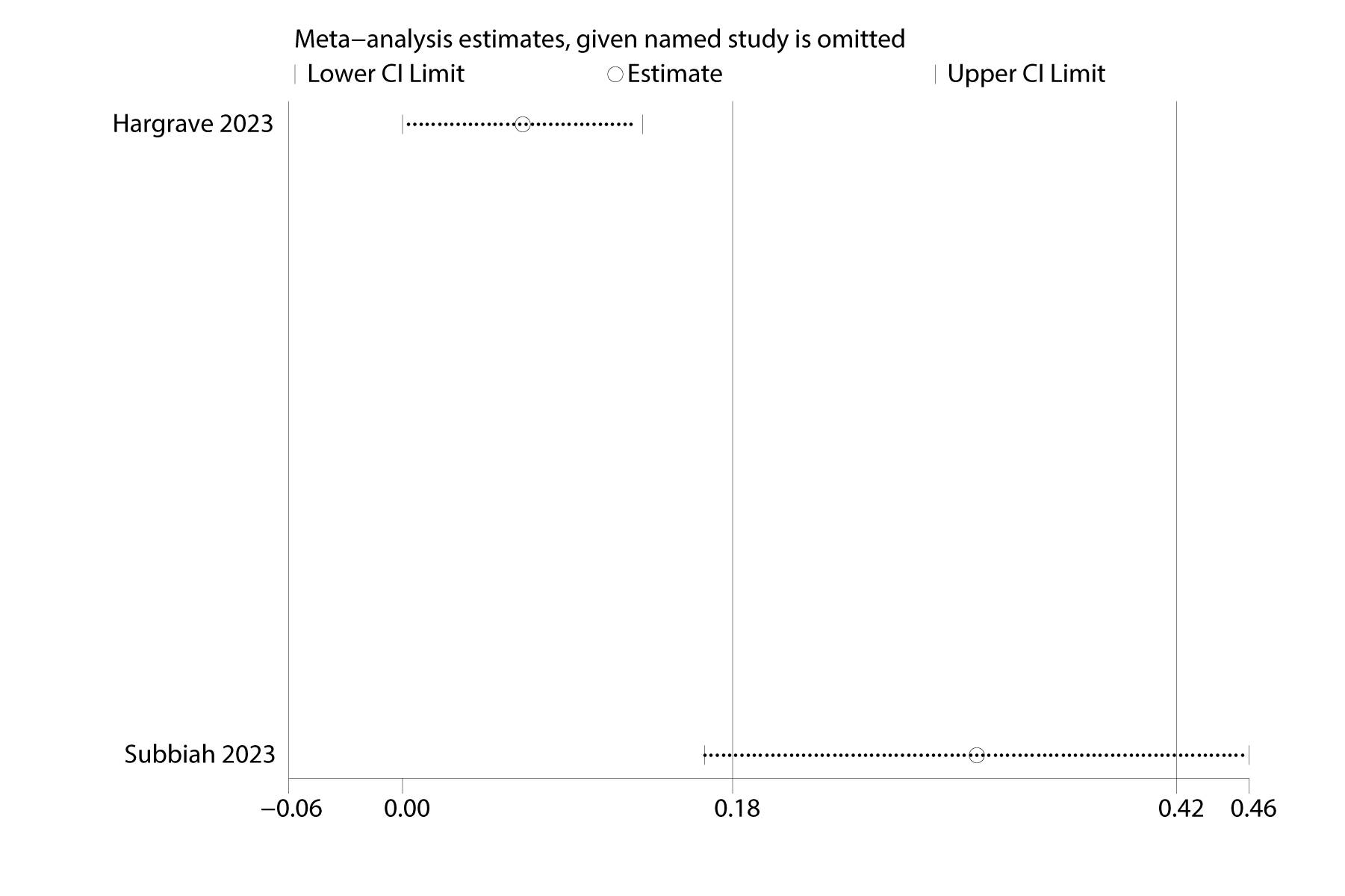

Supplement: Supplementary file 1 — Supplementary file1 Supplementary Figure 1.Forest plot for PFS rate (a) and Subgroups analysis of PFS rate by the intervention (b). Supplementary Figure 2. Forest plot for PR (a) and CR (b). Supplementary Figure 3.Forest plot for ORR (a) and RR (b). Supplementary Figure 4. Subgroups analysis of RR by age (a) and by the intervention (b). Supplementary Figure 5. Subgroups analysis of AEs by age (a) and by the intervention (b). Supplementary Figure 6.The funnel plot for PFS. Supplementary Figure 7.The funnel plot for PFS rate. Supplementary Figure 8.The funnel plot for OS. Supplementary Figure 9.The funnel plot for PR. Supplementary Figure 10.The funnel plot for CR Supplementary Figure 11.The funnel plot for ORR. Supplementary Figure 12.The funnel plot for RR. Supplementary Figure 13.The funnel plot for AEs Supplementary Figure 14.The funnel plot for death events. Supplementary Figure 15.The funnel plot for PFS. Supplementary Figure 16.The funnel plot for PFS rate. Supplementary Figure 17.The funnel plot for OS Supplementary Figure 18.The funnel plot for PR. Supplementary Figure 19.The funnel plot for CR. Supplementary Figure 20.The funnel plot for ORR. Supplementary Figure 21.Sensitivity analysis of RR. Supplementary Figure 22.Sensitivity analysis of AEs. Supplementary Figure 23.Sensitivity analysis of death events (ZIP 1916 KB) [file 10143_2024_2664_MOESM1_ESM.zip › Supplementary Figure 19.jpg]

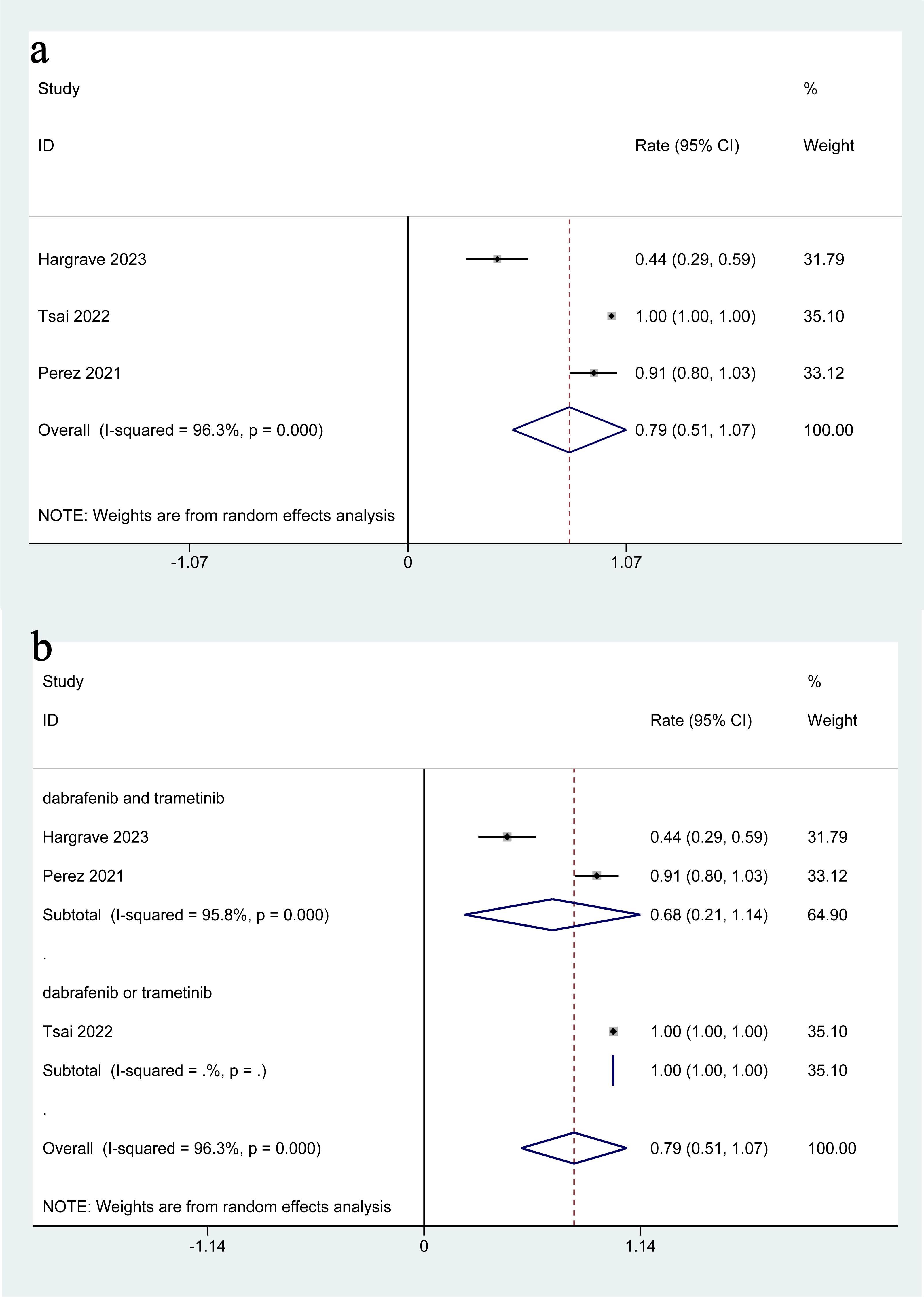

Supplement: Supplementary file 1 — Supplementary file1 Supplementary Figure 1.Forest plot for PFS rate (a) and Subgroups analysis of PFS rate by the intervention (b). Supplementary Figure 2. Forest plot for PR (a) and CR (b). Supplementary Figure 3.Forest plot for ORR (a) and RR (b). Supplementary Figure 4. Subgroups analysis of RR by age (a) and by the intervention (b). Supplementary Figure 5. Subgroups analysis of AEs by age (a) and by the intervention (b). Supplementary Figure 6.The funnel plot for PFS. Supplementary Figure 7.The funnel plot for PFS rate. Supplementary Figure 8.The funnel plot for OS. Supplementary Figure 9.The funnel plot for PR. Supplementary Figure 10.The funnel plot for CR Supplementary Figure 11.The funnel plot for ORR. Supplementary Figure 12.The funnel plot for RR. Supplementary Figure 13.The funnel plot for AEs Supplementary Figure 14.The funnel plot for death events. Supplementary Figure 15.The funnel plot for PFS. Supplementary Figure 16.The funnel plot for PFS rate. Supplementary Figure 17.The funnel plot for OS Supplementary Figure 18.The funnel plot for PR. Supplementary Figure 19.The funnel plot for CR. Supplementary Figure 20.The funnel plot for ORR. Supplementary Figure 21.Sensitivity analysis of RR. Supplementary Figure 22.Sensitivity analysis of AEs. Supplementary Figure 23.Sensitivity analysis of death events (ZIP 1916 KB) [file 10143_2024_2664_MOESM1_ESM.zip › Supplementary Figure 2.jpg]

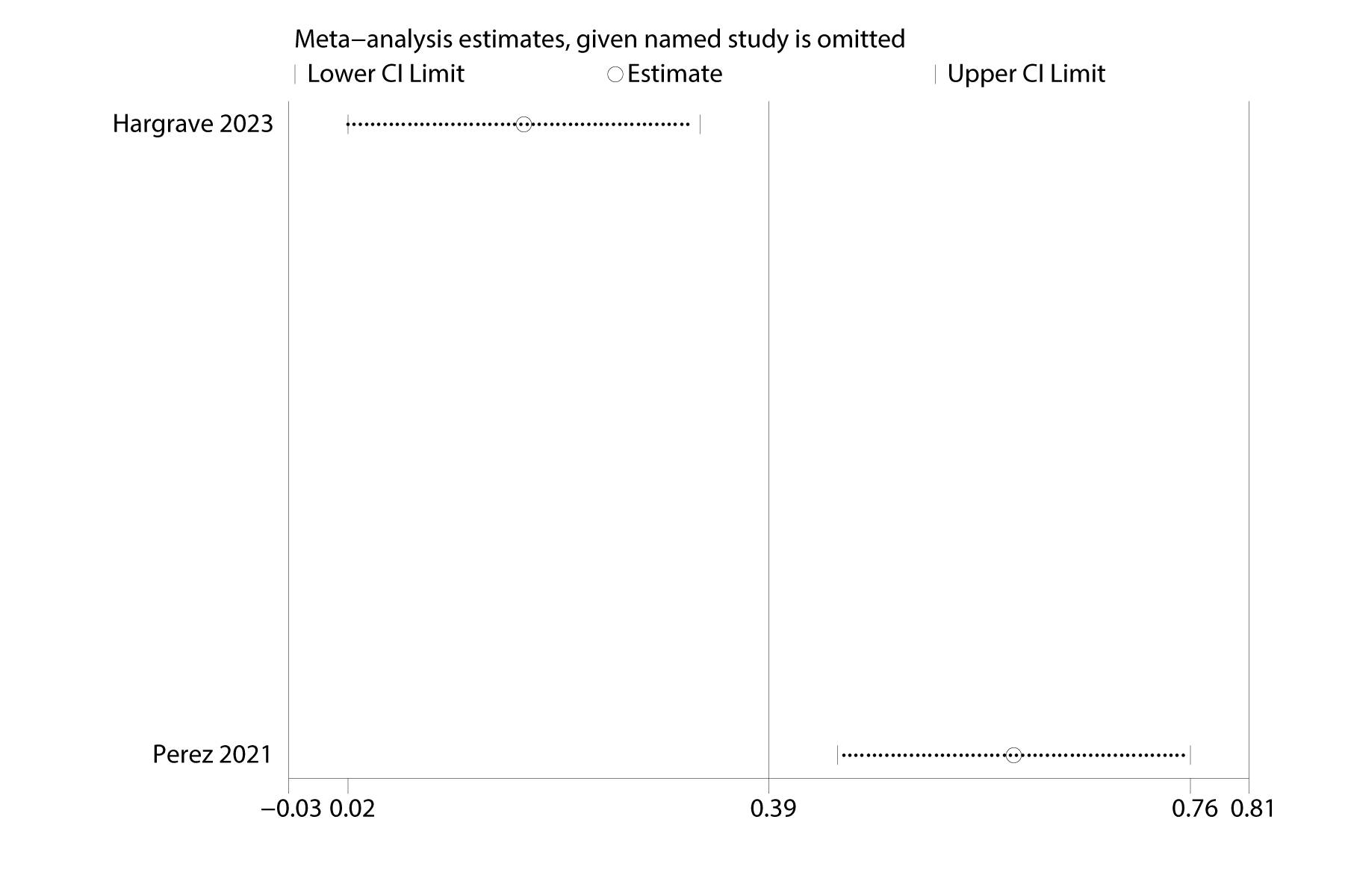

Supplement: Supplementary file 1 — Supplementary file1 Supplementary Figure 1.Forest plot for PFS rate (a) and Subgroups analysis of PFS rate by the intervention (b). Supplementary Figure 2. Forest plot for PR (a) and CR (b). Supplementary Figure 3.Forest plot for ORR (a) and RR (b). Supplementary Figure 4. Subgroups analysis of RR by age (a) and by the intervention (b). Supplementary Figure 5. Subgroups analysis of AEs by age (a) and by the intervention (b). Supplementary Figure 6.The funnel plot for PFS. Supplementary Figure 7.The funnel plot for PFS rate. Supplementary Figure 8.The funnel plot for OS. Supplementary Figure 9.The funnel plot for PR. Supplementary Figure 10.The funnel plot for CR Supplementary Figure 11.The funnel plot for ORR. Supplementary Figure 12.The funnel plot for RR. Supplementary Figure 13.The funnel plot for AEs Supplementary Figure 14.The funnel plot for death events. Supplementary Figure 15.The funnel plot for PFS. Supplementary Figure 16.The funnel plot for PFS rate. Supplementary Figure 17.The funnel plot for OS Supplementary Figure 18.The funnel plot for PR. Supplementary Figure 19.The funnel plot for CR. Supplementary Figure 20.The funnel plot for ORR. Supplementary Figure 21.Sensitivity analysis of RR. Supplementary Figure 22.Sensitivity analysis of AEs. Supplementary Figure 23.Sensitivity analysis of death events (ZIP 1916 KB) [file 10143_2024_2664_MOESM1_ESM.zip › Supplementary Figure 20.jpg]

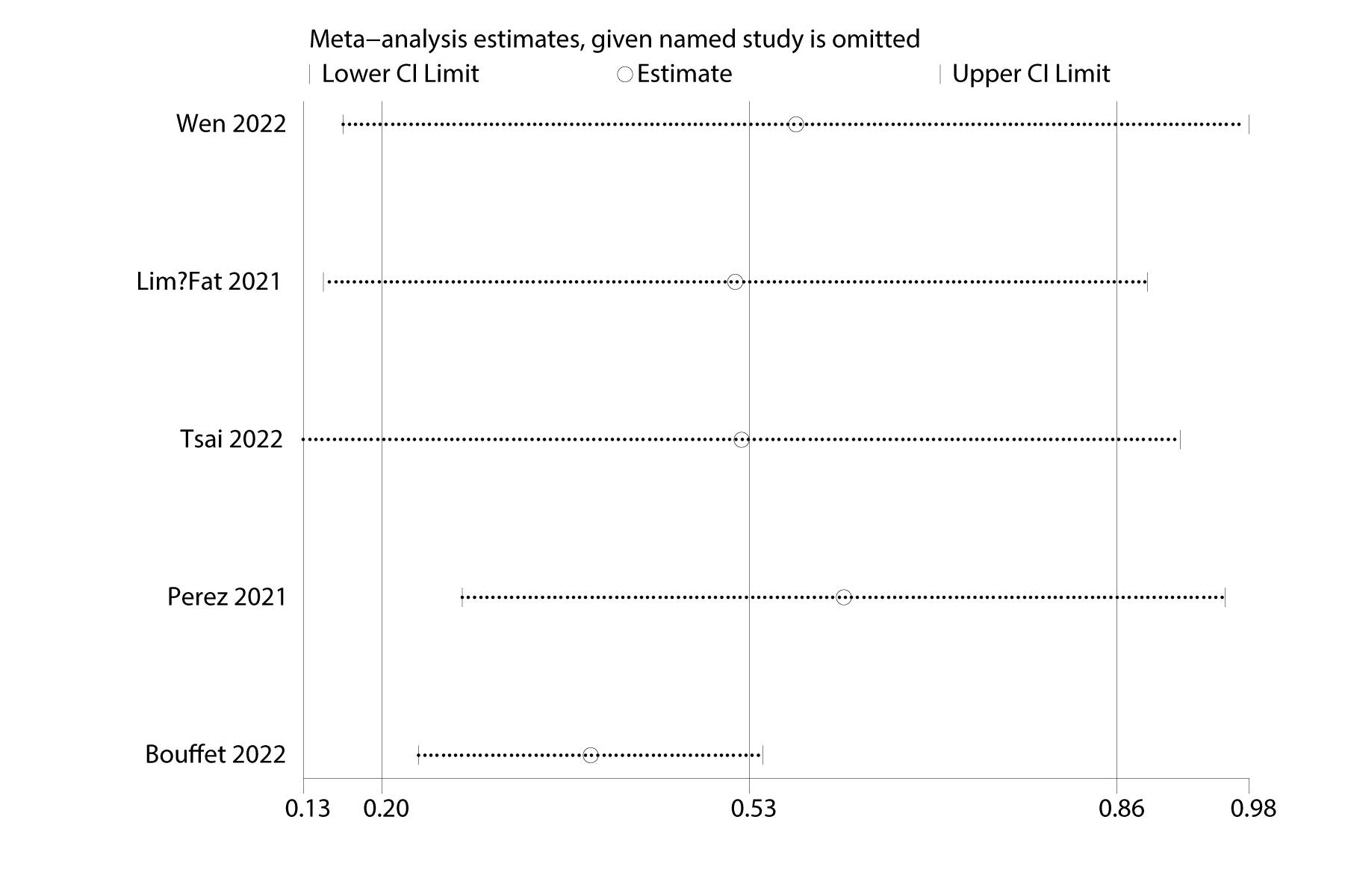

Supplement: Supplementary file 1 — Supplementary file1 Supplementary Figure 1.Forest plot for PFS rate (a) and Subgroups analysis of PFS rate by the intervention (b). Supplementary Figure 2. Forest plot for PR (a) and CR (b). Supplementary Figure 3.Forest plot for ORR (a) and RR (b). Supplementary Figure 4. Subgroups analysis of RR by age (a) and by the intervention (b). Supplementary Figure 5. Subgroups analysis of AEs by age (a) and by the intervention (b). Supplementary Figure 6.The funnel plot for PFS. Supplementary Figure 7.The funnel plot for PFS rate. Supplementary Figure 8.The funnel plot for OS. Supplementary Figure 9.The funnel plot for PR. Supplementary Figure 10.The funnel plot for CR Supplementary Figure 11.The funnel plot for ORR. Supplementary Figure 12.The funnel plot for RR. Supplementary Figure 13.The funnel plot for AEs Supplementary Figure 14.The funnel plot for death events. Supplementary Figure 15.The funnel plot for PFS. Supplementary Figure 16.The funnel plot for PFS rate. Supplementary Figure 17.The funnel plot for OS Supplementary Figure 18.The funnel plot for PR. Supplementary Figure 19.The funnel plot for CR. Supplementary Figure 20.The funnel plot for ORR. Supplementary Figure 21.Sensitivity analysis of RR. Supplementary Figure 22.Sensitivity analysis of AEs. Supplementary Figure 23.Sensitivity analysis of death events (ZIP 1916 KB) [file 10143_2024_2664_MOESM1_ESM.zip › Supplementary Figure 21.jpg]

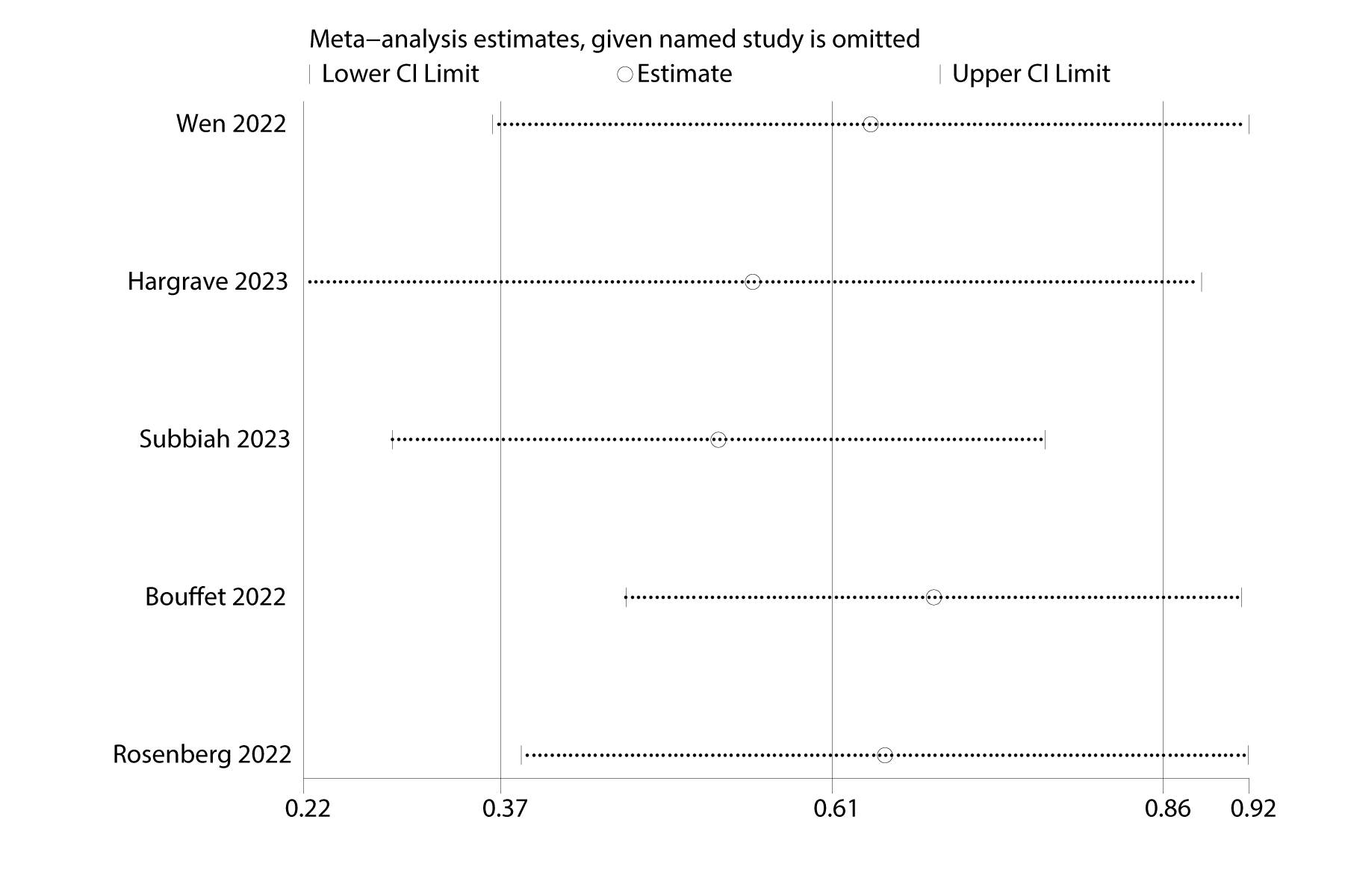

Supplement: Supplementary file 1 — Supplementary file1 Supplementary Figure 1.Forest plot for PFS rate (a) and Subgroups analysis of PFS rate by the intervention (b). Supplementary Figure 2. Forest plot for PR (a) and CR (b). Supplementary Figure 3.Forest plot for ORR (a) and RR (b). Supplementary Figure 4. Subgroups analysis of RR by age (a) and by the intervention (b). Supplementary Figure 5. Subgroups analysis of AEs by age (a) and by the intervention (b). Supplementary Figure 6.The funnel plot for PFS. Supplementary Figure 7.The funnel plot for PFS rate. Supplementary Figure 8.The funnel plot for OS. Supplementary Figure 9.The funnel plot for PR. Supplementary Figure 10.The funnel plot for CR Supplementary Figure 11.The funnel plot for ORR. Supplementary Figure 12.The funnel plot for RR. Supplementary Figure 13.The funnel plot for AEs Supplementary Figure 14.The funnel plot for death events. Supplementary Figure 15.The funnel plot for PFS. Supplementary Figure 16.The funnel plot for PFS rate. Supplementary Figure 17.The funnel plot for OS Supplementary Figure 18.The funnel plot for PR. Supplementary Figure 19.The funnel plot for CR. Supplementary Figure 20.The funnel plot for ORR. Supplementary Figure 21.Sensitivity analysis of RR. Supplementary Figure 22.Sensitivity analysis of AEs. Supplementary Figure 23.Sensitivity analysis of death events (ZIP 1916 KB) [file 10143_2024_2664_MOESM1_ESM.zip › Supplementary Figure 22.jpg]

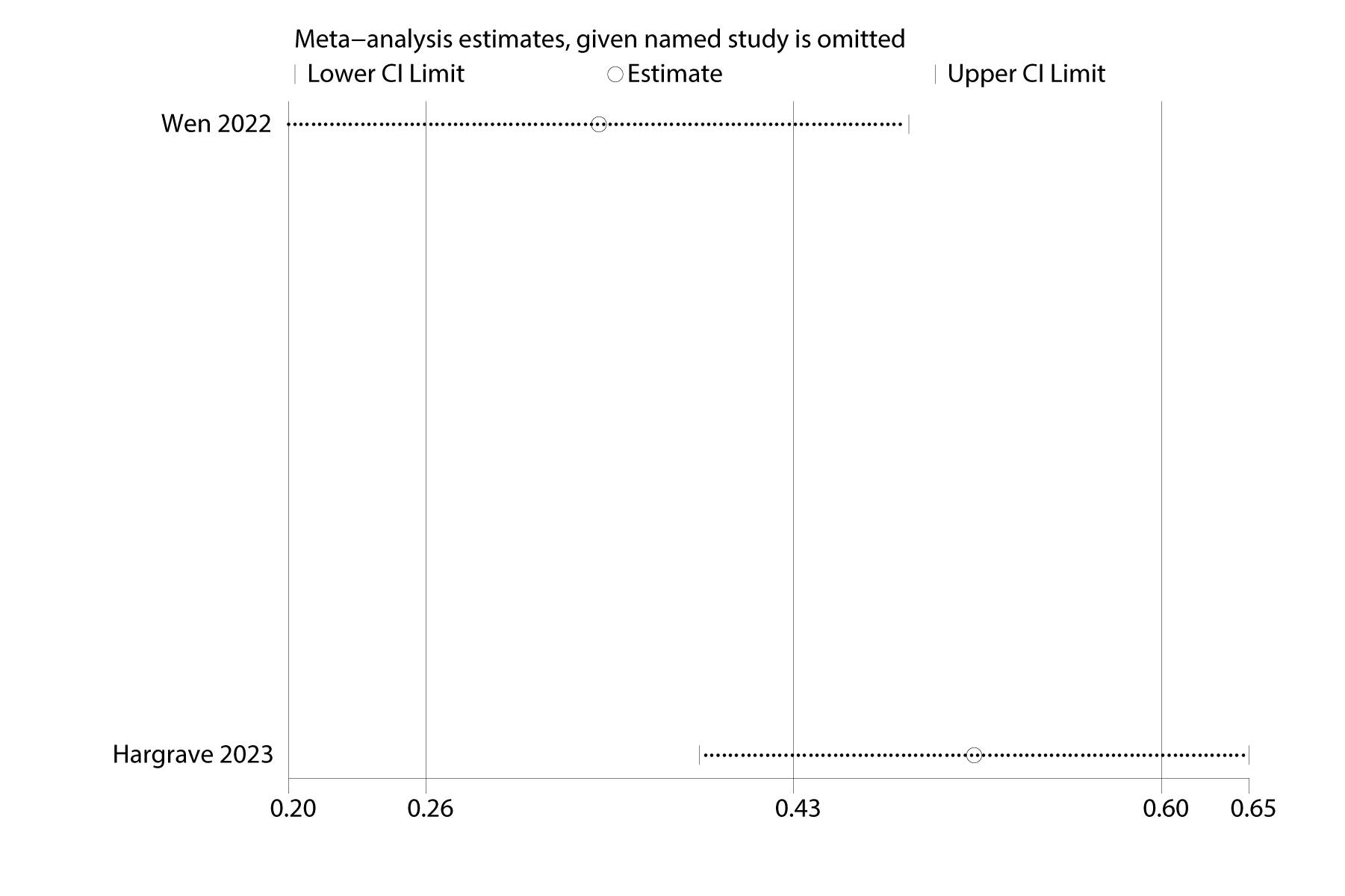

Supplement: Supplementary file 1 — Supplementary file1 Supplementary Figure 1.Forest plot for PFS rate (a) and Subgroups analysis of PFS rate by the intervention (b). Supplementary Figure 2. Forest plot for PR (a) and CR (b). Supplementary Figure 3.Forest plot for ORR (a) and RR (b). Supplementary Figure 4. Subgroups analysis of RR by age (a) and by the intervention (b). Supplementary Figure 5. Subgroups analysis of AEs by age (a) and by the intervention (b). Supplementary Figure 6.The funnel plot for PFS. Supplementary Figure 7.The funnel plot for PFS rate. Supplementary Figure 8.The funnel plot for OS. Supplementary Figure 9.The funnel plot for PR. Supplementary Figure 10.The funnel plot for CR Supplementary Figure 11.The funnel plot for ORR. Supplementary Figure 12.The funnel plot for RR. Supplementary Figure 13.The funnel plot for AEs Supplementary Figure 14.The funnel plot for death events. Supplementary Figure 15.The funnel plot for PFS. Supplementary Figure 16.The funnel plot for PFS rate. Supplementary Figure 17.The funnel plot for OS Supplementary Figure 18.The funnel plot for PR. Supplementary Figure 19.The funnel plot for CR. Supplementary Figure 20.The funnel plot for ORR. Supplementary Figure 21.Sensitivity analysis of RR. Supplementary Figure 22.Sensitivity analysis of AEs. Supplementary Figure 23.Sensitivity analysis of death events (ZIP 1916 KB) [file 10143_2024_2664_MOESM1_ESM.zip › Supplementary Figure 23.jpg]

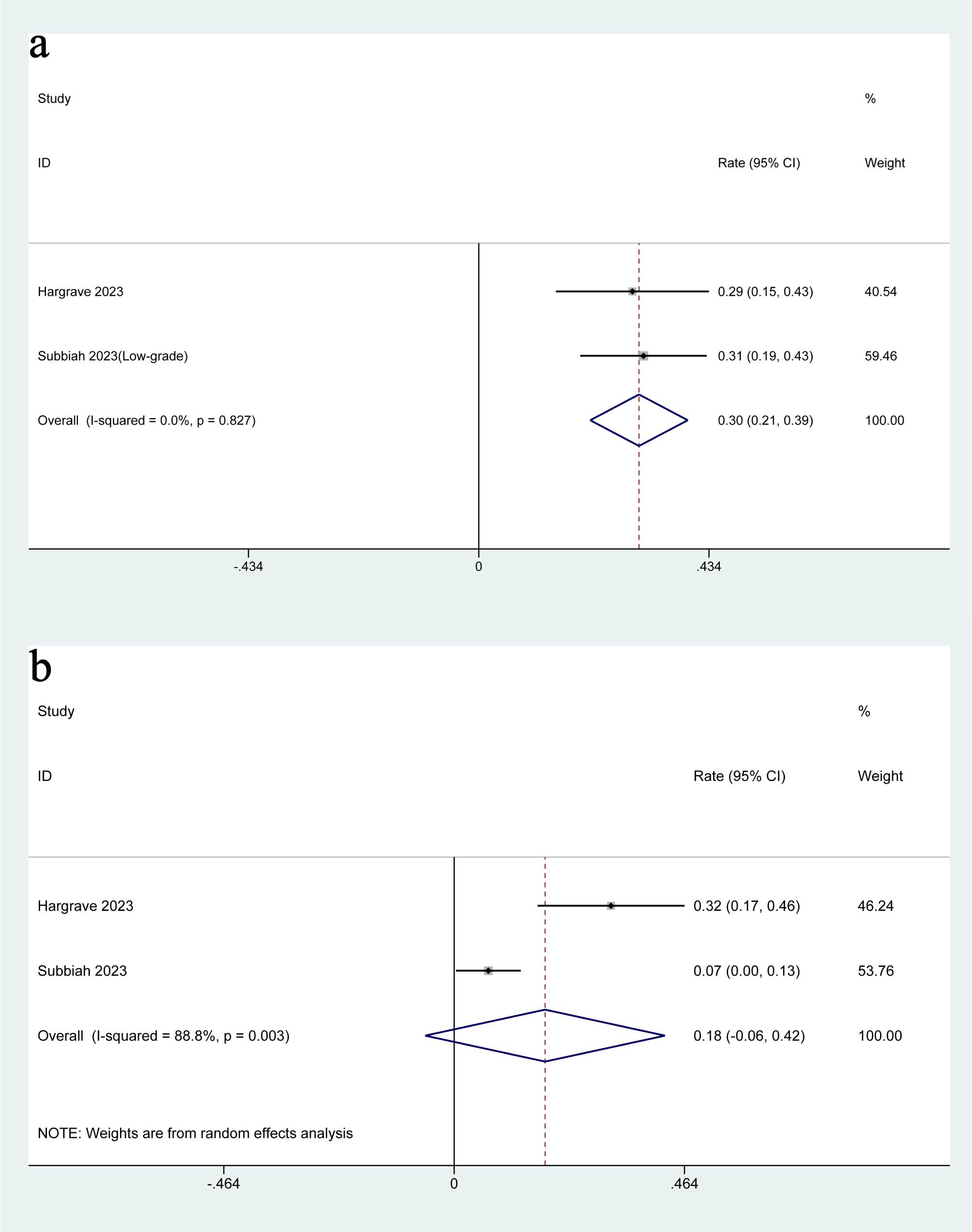

Supplement: Supplementary file 1 — Supplementary file1 Supplementary Figure 1.Forest plot for PFS rate (a) and Subgroups analysis of PFS rate by the intervention (b). Supplementary Figure 2. Forest plot for PR (a) and CR (b). Supplementary Figure 3.Forest plot for ORR (a) and RR (b). Supplementary Figure 4. Subgroups analysis of RR by age (a) and by the intervention (b). Supplementary Figure 5. Subgroups analysis of AEs by age (a) and by the intervention (b). Supplementary Figure 6.The funnel plot for PFS. Supplementary Figure 7.The funnel plot for PFS rate. Supplementary Figure 8.The funnel plot for OS. Supplementary Figure 9.The funnel plot for PR. Supplementary Figure 10.The funnel plot for CR Supplementary Figure 11.The funnel plot for ORR. Supplementary Figure 12.The funnel plot for RR. Supplementary Figure 13.The funnel plot for AEs Supplementary Figure 14.The funnel plot for death events. Supplementary Figure 15.The funnel plot for PFS. Supplementary Figure 16.The funnel plot for PFS rate. Supplementary Figure 17.The funnel plot for OS Supplementary Figure 18.The funnel plot for PR. Supplementary Figure 19.The funnel plot for CR. Supplementary Figure 20.The funnel plot for ORR. Supplementary Figure 21.Sensitivity analysis of RR. Supplementary Figure 22.Sensitivity analysis of AEs. Supplementary Figure 23.Sensitivity analysis of death events (ZIP 1916 KB) [file 10143_2024_2664_MOESM1_ESM.zip › Supplementary Figure 3.jpg]

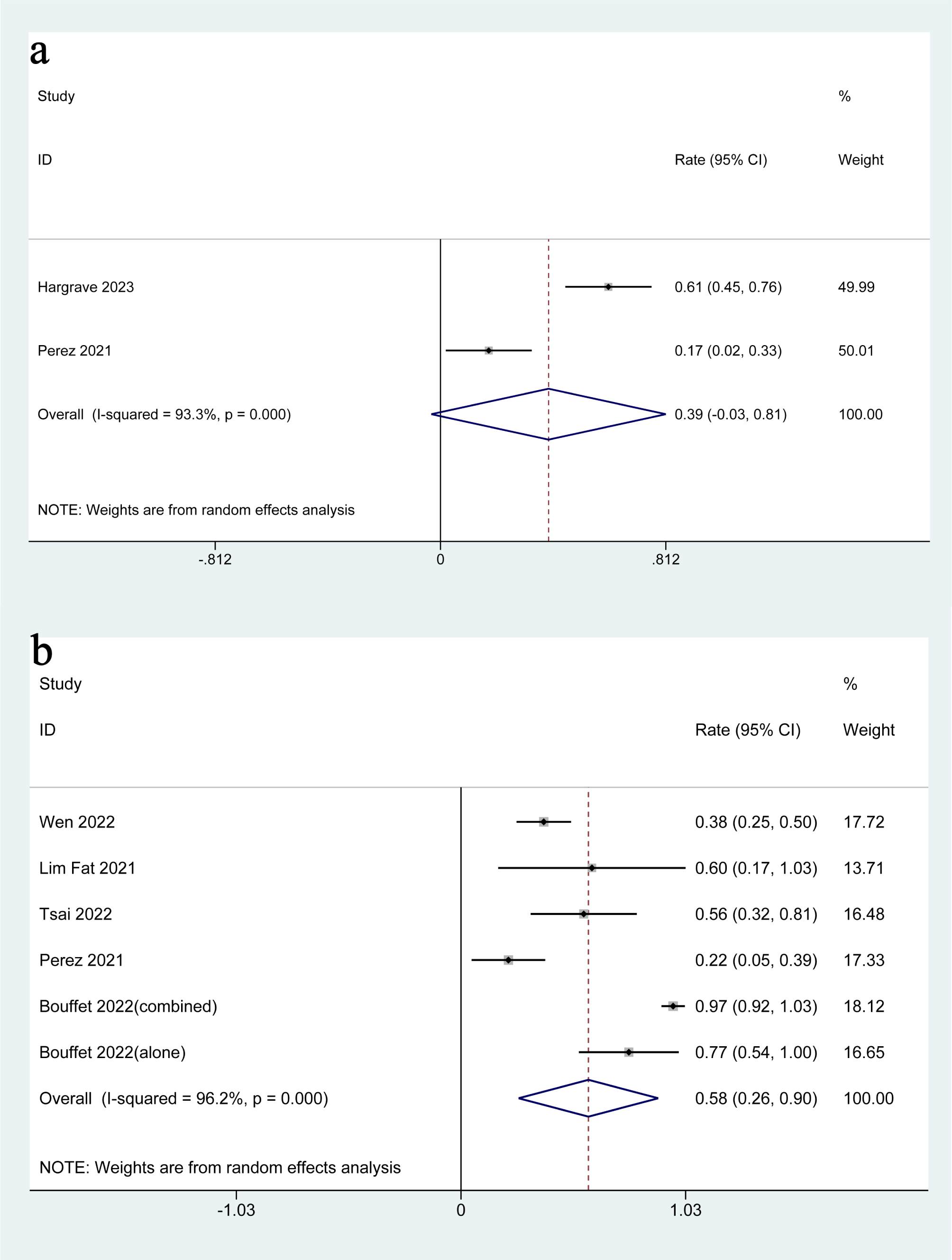

Supplement: Supplementary file 1 — Supplementary file1 Supplementary Figure 1.Forest plot for PFS rate (a) and Subgroups analysis of PFS rate by the intervention (b). Supplementary Figure 2. Forest plot for PR (a) and CR (b). Supplementary Figure 3.Forest plot for ORR (a) and RR (b). Supplementary Figure 4. Subgroups analysis of RR by age (a) and by the intervention (b). Supplementary Figure 5. Subgroups analysis of AEs by age (a) and by the intervention (b). Supplementary Figure 6.The funnel plot for PFS. Supplementary Figure 7.The funnel plot for PFS rate. Supplementary Figure 8.The funnel plot for OS. Supplementary Figure 9.The funnel plot for PR. Supplementary Figure 10.The funnel plot for CR Supplementary Figure 11.The funnel plot for ORR. Supplementary Figure 12.The funnel plot for RR. Supplementary Figure 13.The funnel plot for AEs Supplementary Figure 14.The funnel plot for death events. Supplementary Figure 15.The funnel plot for PFS. Supplementary Figure 16.The funnel plot for PFS rate. Supplementary Figure 17.The funnel plot for OS Supplementary Figure 18.The funnel plot for PR. Supplementary Figure 19.The funnel plot for CR. Supplementary Figure 20.The funnel plot for ORR. Supplementary Figure 21.Sensitivity analysis of RR. Supplementary Figure 22.Sensitivity analysis of AEs. Supplementary Figure 23.Sensitivity analysis of death events (ZIP 1916 KB) [file 10143_2024_2664_MOESM1_ESM.zip › Supplementary Figure 4.jpg]

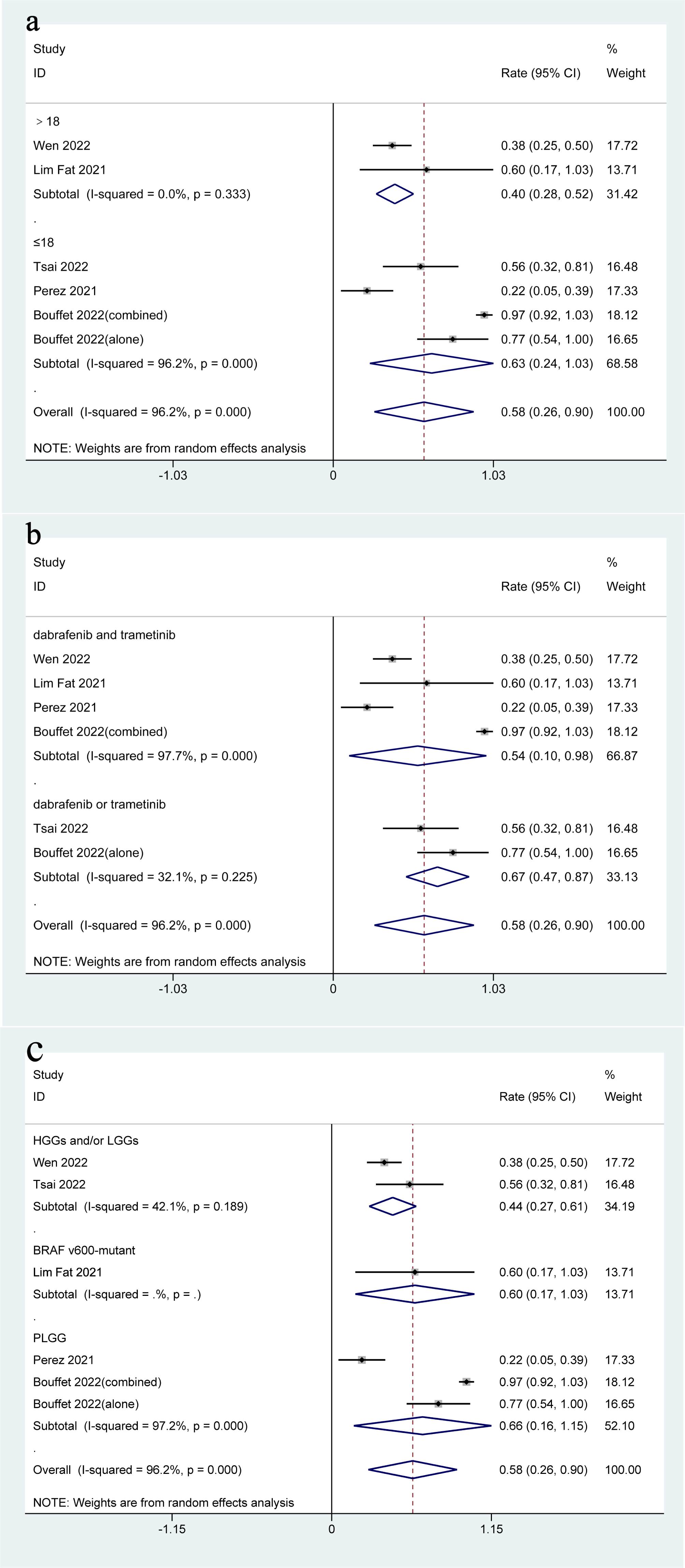

Supplement: Supplementary file 1 — Supplementary file1 Supplementary Figure 1.Forest plot for PFS rate (a) and Subgroups analysis of PFS rate by the intervention (b). Supplementary Figure 2. Forest plot for PR (a) and CR (b). Supplementary Figure 3.Forest plot for ORR (a) and RR (b). Supplementary Figure 4. Subgroups analysis of RR by age (a) and by the intervention (b). Supplementary Figure 5. Subgroups analysis of AEs by age (a) and by the intervention (b). Supplementary Figure 6.The funnel plot for PFS. Supplementary Figure 7.The funnel plot for PFS rate. Supplementary Figure 8.The funnel plot for OS. Supplementary Figure 9.The funnel plot for PR. Supplementary Figure 10.The funnel plot for CR Supplementary Figure 11.The funnel plot for ORR. Supplementary Figure 12.The funnel plot for RR. Supplementary Figure 13.The funnel plot for AEs Supplementary Figure 14.The funnel plot for death events. Supplementary Figure 15.The funnel plot for PFS. Supplementary Figure 16.The funnel plot for PFS rate. Supplementary Figure 17.The funnel plot for OS Supplementary Figure 18.The funnel plot for PR. Supplementary Figure 19.The funnel plot for CR. Supplementary Figure 20.The funnel plot for ORR. Supplementary Figure 21.Sensitivity analysis of RR. Supplementary Figure 22.Sensitivity analysis of AEs. Supplementary Figure 23.Sensitivity analysis of death events (ZIP 1916 KB) [file 10143_2024_2664_MOESM1_ESM.zip › Supplementary Figure 5.jpg]

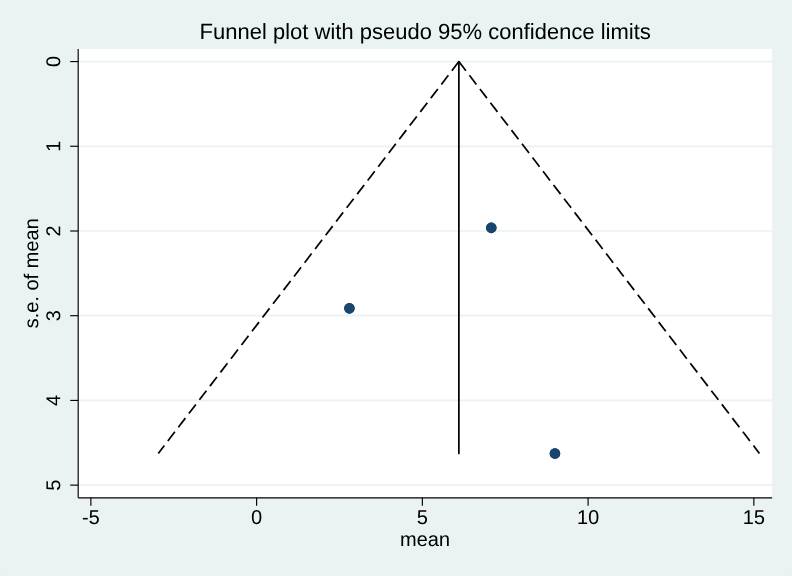

Supplement: Supplementary file 1 — Supplementary file1 Supplementary Figure 1.Forest plot for PFS rate (a) and Subgroups analysis of PFS rate by the intervention (b). Supplementary Figure 2. Forest plot for PR (a) and CR (b). Supplementary Figure 3.Forest plot for ORR (a) and RR (b). Supplementary Figure 4. Subgroups analysis of RR by age (a) and by the intervention (b). Supplementary Figure 5. Subgroups analysis of AEs by age (a) and by the intervention (b). Supplementary Figure 6.The funnel plot for PFS. Supplementary Figure 7.The funnel plot for PFS rate. Supplementary Figure 8.The funnel plot for OS. Supplementary Figure 9.The funnel plot for PR. Supplementary Figure 10.The funnel plot for CR Supplementary Figure 11.The funnel plot for ORR. Supplementary Figure 12.The funnel plot for RR. Supplementary Figure 13.The funnel plot for AEs Supplementary Figure 14.The funnel plot for death events. Supplementary Figure 15.The funnel plot for PFS. Supplementary Figure 16.The funnel plot for PFS rate. Supplementary Figure 17.The funnel plot for OS Supplementary Figure 18.The funnel plot for PR. Supplementary Figure 19.The funnel plot for CR. Supplementary Figure 20.The funnel plot for ORR. Supplementary Figure 21.Sensitivity analysis of RR. Supplementary Figure 22.Sensitivity analysis of AEs. Supplementary Figure 23.Sensitivity analysis of death events (ZIP 1916 KB) [file 10143_2024_2664_MOESM1_ESM.zip › Supplementary Figure 6.jpg]

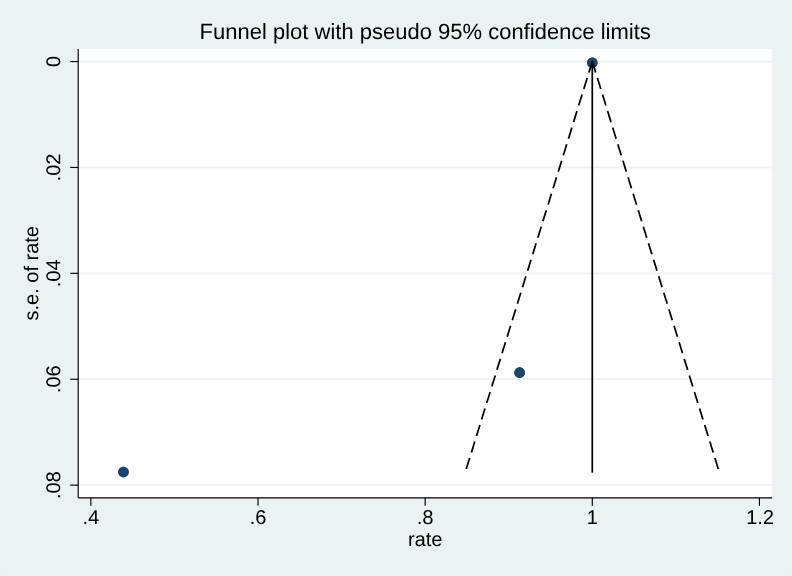

Supplement: Supplementary file 1 — Supplementary file1 Supplementary Figure 1.Forest plot for PFS rate (a) and Subgroups analysis of PFS rate by the intervention (b). Supplementary Figure 2. Forest plot for PR (a) and CR (b). Supplementary Figure 3.Forest plot for ORR (a) and RR (b). Supplementary Figure 4. Subgroups analysis of RR by age (a) and by the intervention (b). Supplementary Figure 5. Subgroups analysis of AEs by age (a) and by the intervention (b). Supplementary Figure 6.The funnel plot for PFS. Supplementary Figure 7.The funnel plot for PFS rate. Supplementary Figure 8.The funnel plot for OS. Supplementary Figure 9.The funnel plot for PR. Supplementary Figure 10.The funnel plot for CR Supplementary Figure 11.The funnel plot for ORR. Supplementary Figure 12.The funnel plot for RR. Supplementary Figure 13.The funnel plot for AEs Supplementary Figure 14.The funnel plot for death events. Supplementary Figure 15.The funnel plot for PFS. Supplementary Figure 16.The funnel plot for PFS rate. Supplementary Figure 17.The funnel plot for OS Supplementary Figure 18.The funnel plot for PR. Supplementary Figure 19.The funnel plot for CR. Supplementary Figure 20.The funnel plot for ORR. Supplementary Figure 21.Sensitivity analysis of RR. Supplementary Figure 22.Sensitivity analysis of AEs. Supplementary Figure 23.Sensitivity analysis of death events (ZIP 1916 KB) [file 10143_2024_2664_MOESM1_ESM.zip › Supplementary Figure 7.jpg]

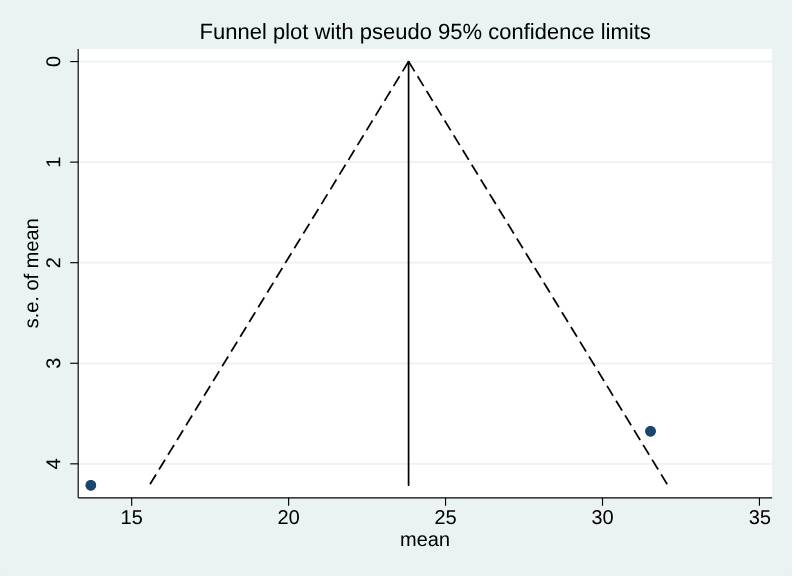

Supplement: Supplementary file 1 — Supplementary file1 Supplementary Figure 1.Forest plot for PFS rate (a) and Subgroups analysis of PFS rate by the intervention (b). Supplementary Figure 2. Forest plot for PR (a) and CR (b). Supplementary Figure 3.Forest plot for ORR (a) and RR (b). Supplementary Figure 4. Subgroups analysis of RR by age (a) and by the intervention (b). Supplementary Figure 5. Subgroups analysis of AEs by age (a) and by the intervention (b). Supplementary Figure 6.The funnel plot for PFS. Supplementary Figure 7.The funnel plot for PFS rate. Supplementary Figure 8.The funnel plot for OS. Supplementary Figure 9.The funnel plot for PR. Supplementary Figure 10.The funnel plot for CR Supplementary Figure 11.The funnel plot for ORR. Supplementary Figure 12.The funnel plot for RR. Supplementary Figure 13.The funnel plot for AEs Supplementary Figure 14.The funnel plot for death events. Supplementary Figure 15.The funnel plot for PFS. Supplementary Figure 16.The funnel plot for PFS rate. Supplementary Figure 17.The funnel plot for OS Supplementary Figure 18.The funnel plot for PR. Supplementary Figure 19.The funnel plot for CR. Supplementary Figure 20.The funnel plot for ORR. Supplementary Figure 21.Sensitivity analysis of RR. Supplementary Figure 22.Sensitivity analysis of AEs. Supplementary Figure 23.Sensitivity analysis of death events (ZIP 1916 KB) [file 10143_2024_2664_MOESM1_ESM.zip › Supplementary Figure 8.jpg]

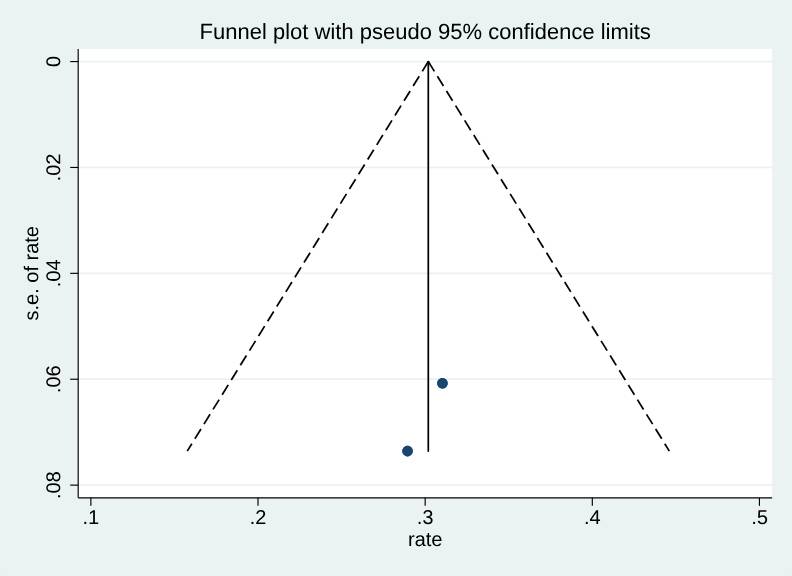

Supplement: Supplementary file 1 — Supplementary file1 Supplementary Figure 1.Forest plot for PFS rate (a) and Subgroups analysis of PFS rate by the intervention (b). Supplementary Figure 2. Forest plot for PR (a) and CR (b). Supplementary Figure 3.Forest plot for ORR (a) and RR (b). Supplementary Figure 4. Subgroups analysis of RR by age (a) and by the intervention (b). Supplementary Figure 5. Subgroups analysis of AEs by age (a) and by the intervention (b). Supplementary Figure 6.The funnel plot for PFS. Supplementary Figure 7.The funnel plot for PFS rate. Supplementary Figure 8.The funnel plot for OS. Supplementary Figure 9.The funnel plot for PR. Supplementary Figure 10.The funnel plot for CR Supplementary Figure 11.The funnel plot for ORR. Supplementary Figure 12.The funnel plot for RR. Supplementary Figure 13.The funnel plot for AEs Supplementary Figure 14.The funnel plot for death events. Supplementary Figure 15.The funnel plot for PFS. Supplementary Figure 16.The funnel plot for PFS rate. Supplementary Figure 17.The funnel plot for OS Supplementary Figure 18.The funnel plot for PR. Supplementary Figure 19.The funnel plot for CR. Supplementary Figure 20.The funnel plot for ORR. Supplementary Figure 21.Sensitivity analysis of RR. Supplementary Figure 22.Sensitivity analysis of AEs. Supplementary Figure 23.Sensitivity analysis of death events (ZIP 1916 KB) [file 10143_2024_2664_MOESM1_ESM.zip › Supplementary Figure 9.jpg]
