# Supplementary material for: The effects of dabrafenib and/or trametinib treatment in Braf V600-mutant glioma: a systematic review and meta-analysis
Source: Neurosurg Rev. 2024 Aug 22;47(1):458. doi: 10.1007/s10143-024-02664-x (PMC11341626; doi:10.1007/s10143-024-02664-x)
Supplement: Supplementary file 2 — Supplementary file2 Supplementary Table 1.Search strategy. (DOCX 21 KB) [file 10143_2024_2664_MOESM2_ESM.docx]

**Search strategy of PubMed**

| NO. | Search Details | Results |
| --- | --- | --- |
| #7 | (#1 OR #4) AND (#2 OR #5) AND (#3 OR #6) | 310 |
| #6 | (((((((((((((((((Gliomas) OR (Glial Cell Tumors)) OR (Glial Cell Tumor)) OR (Mixed Glioma)) OR (Mixed Gliomas)) OR (Malignant Glioma)) OR (Malignant Gliomas)) OR (brain glioma)) OR (cerebral glioma)) OR (ganglioglioma)) OR (glia tumor)) OR (glia tumour)) OR (glial tumor)) OR (glial tumour)) OR (high grade glioma)) OR (low grade glioma)) OR (recurrent glioma)) OR (glioma) | 139,528 |
| #5 | ((((((((((((((gsk 1120212) OR (gsk 1120212b)) OR (gsk1120212)) OR (gsk1120212b)) OR (jtp 74057)) OR (jtp74057)) OR (mekinist)) OR (snr 1611)) OR (snr1611)) OR (tmt 212)) OR (tmt212)) OR (trametinib dimethyl sulfoxide)) OR (trametinib)) OR (JTP-74057)) OR (GSK-1120212) | 37,960 |
| #4 | (((((((((((((((dabrafenib mesilate) OR (dabrafenib mesylate)) OR (drb 436)) OR (drb436)) OR (gsk 2118436)) OR (gsk 2118436a)) OR (gsk 2118436b)) OR (gsk2118436)) OR (gsk2118436a)) OR (gsk2118436b)) OR (taffiner)) OR (tafinlar)) OR (tafinra)) OR (tafinrar)) OR (dabrafenib)) OR (GSK-2118436) | 37,549 |
| #3 | "Glioma"[Mesh] | 99,640 |
| #2 | "trametinib" [Supplementary Concept] | 961 |
| #1 | "dabrafenib" [Supplementary Concept] | 822 |

**Search strategy of EMBASE**

| No. | Query | Results |
| --- | --- | --- |
| #7 | (#1 OR #2) AND (#3 OR #4) AND (#5 OR #6) | 347 |
| #6 | 'gliomas':ti,ab,kw OR 'glial cell tumors':ti,ab,kw OR 'glial cell tumor':ti,ab,kw OR 'mixed glioma':ti,ab,kw OR 'mixed gliomas':ti,ab,kw OR 'malignant glioma':ti,ab,kw OR 'malignant gliomas':ti,ab,kw OR 'brain glioma':ti,ab,kw OR 'cerebral glioma':ti,ab,kw OR 'ganglioglioma':ti,ab,kw OR 'glia tumor':ti,ab,kw OR 'glia tumour':ti,ab,kw OR 'glial tumor':ti,ab,kw OR 'glial tumour':ti,ab,kw OR 'high grade glioma':ti,ab,kw OR 'low grade glioma':ti,ab,kw OR 'recurrent glioma':ti,ab,kw OR 'glioma':ti,ab,kw | 105079 |
| #5 | 'glioma'/exp | 180414 |
| #4 | 'gsk 1120212':ti,ab,kw OR 'gsk 1120212b':ti,ab,kw OR 'gsk1120212':ti,ab,kw OR 'gsk1120212b':ti,ab,kw OR 'jtp 74057':ti,ab,kw OR 'jtp74057':ti,ab,kw OR 'mekinist':ti,ab,kw OR 'snr 1611':ti,ab,kw OR 'snr1611':ti,ab,kw OR 'tmt 212':ti,ab,kw OR 'tmt212':ti,ab,kw OR 'trametinib dimethyl sulfoxide':ti,ab,kw OR 'trametinib':ti,ab,kw OR 'jtp-74057':ti,ab,kw OR 'gsk-1120212':ti,ab,kw | 4274 |
| #3 | 'trametinib'/exp | 9175 |
| #2 | 'dabrafenib mesylate':ti,ab,kw OR 'drb 436':ti,ab,kw OR 'drb436':ti,ab,kw OR 'gsk 2118436':ti,ab,kw OR 'gsk 2118436a':ti,ab,kw OR 'gsk 2118436b':ti,ab,kw OR 'gsk2118436':ti,ab,kw OR 'gsk2118436a':ti,ab,kw OR 'gsk2118436b':ti,ab,kw OR 'taffiner':ti,ab,kw OR 'tafinlar':ti,ab,kw OR 'tafinra':ti,ab,kw OR 'tafinrar':ti,ab,kw OR 'dabrafenib':ti,ab,kw OR 'gsk-2118436':ti,ab,kw | 3186 |
| #1 | 'dabrafenib'/exp | 7101 |

**Search strategy of Cochrane Library**

| NO. | Search deatiles | Hits |
| --- | --- | --- |
| #1 | MeSH descriptor: [Glioma] explode all trees | 2077 |
| #2 | (dabrafenib mesylate):ti,ab,kw OR (dabrafenib mesylate):ti,ab,kw OR (drb 436):ti,ab,kw OR (drb436):ti,ab,kw OR (gsk 2118436):ti,ab,kw OR (gsk 2118436a):ti,ab,kw OR (gsk 2118436b):ti,ab,kw OR (gsk2118436):ti,ab,kw OR (gsk2118436a):ti,ab,kw OR (gsk2118436b):ti,ab,kw OR (taffiner):ti,ab,kw OR (tafinlar):ti,ab,kw OR (tafinra):ti,ab,kw OR (tafinrar):ti,ab,kw OR (dabrafenib):ti,ab,kw OR (GSK-2118436):ti,ab,kw | 291 |
| #3 | (gsk 1120212):ti,ab,kw OR (gsk 1120212b):ti,ab,kw OR (gsk1120212):ti,ab,kw OR (gsk1120212b):ti,ab,kw OR (jtp 74057):ti,ab,kw OR (jtp74057):ti,ab,kw OR (mekinist):ti,ab,kw OR (snr 1611):ti,ab,kw OR (snr1611):ti,ab,kw OR (tmt 212):ti,ab,kw OR (tmt212):ti,ab,kw OR (trametinib dimethyl sulfoxide):ti,ab,kw OR (trametinib):ti,ab,kw OR (JTP-74057):ti,ab,kw OR (GSK-1120212):ti,ab,kw | 369 |
| #4 | (Gliomas):ti,ab,kw OR (Glial Cell Tumors):ti,ab,kw OR (Glial Cell Tumor):ti,ab,kw OR (Mixed Glioma):ti,ab,kw OR (Mixed Gliomas):ti,ab,kw OR (Malignant Glioma):ti,ab,kw OR (Malignant Gliomas):ti,ab,kw OR (brain glioma):ti,ab,kw OR (cerebral glioma):ti,ab,kw OR (ganglioglioma):ti,ab,kw OR (glia tumor):ti,ab,kw OR (glia tumour):ti,ab,kw OR (glial tumor):ti,ab,kw OR (glial tumour):ti,ab,kw OR (high grade glioma):ti,ab,kw OR (low grade glioma):ti,ab,kw OR (recurrent glioma):ti,ab,kw OR (glioma):ti,ab,kw | 2118 |
| #5 | (#1 OR #4) AND #2 AND #3 | 4 |

**Search strategy of web of science**

| NO. | Search deatiles | Hits |
| --- | --- | --- |
| #1 | ((((((((((((((TS=(dabrafenib mesilate) OR TS=(dabrafenib mesylate)) OR TS=(drb 436)) OR TS=(drb436)) OR TS=(gsk 2118436)) OR TS=(gsk 2118436a)) OR TS=(gsk 2118436b)) OR TS=(gsk2118436)) OR TS=(gsk2118436a)) OR TS=(gsk2118436b)) OR TS=(taffiner)) OR TS=(tafinlar)) OR TS=(tafinra)) OR TS=(tafinrar)) OR TS=(dabrafenib)) OR TS=(GSK-2118436) | 2894 |
| #2 | (((((((((((((TS=(gsk 1120212) OR TS=(gsk 1120212b)) OR TS=(gsk1120212)) OR TS=(gsk1120212b)) OR TS=(jtp 74057)) OR TS=(jtp74057)) OR TS=(mekinist)) OR TS=(snr 1611)) OR TS=(snr1611)) OR TS=(tmt 212)) OR TS=(tmt212)) OR TS=(trametinib dimethyl sulfoxide)) OR TS=(trametinib)) OR TS=(JTP-74057)) OR TS=(GSK-1120212) | 3012 |
| #3 | ((((((((((((((((TS=(Gliomas) OR TS=(Glial Cell Tumors)) OR TS=(Glial Cell Tumor)) OR TS=(Mixed Glioma)) OR TS=(Mixed Gliomas)) OR TS=(Malignant Glioma)) OR TS=(Malignant Gliomas)) OR TS=(brain glioma)) OR TS=(cerebral glioma)) OR TS=(ganglioglioma)) OR TS=(glia tumor)) OR TS=(glia tumour)) OR TS=(glial tumor)) OR TS=(glial tumour)) OR TS=(high grade glioma)) OR TS=(low grade glioma)) OR TS=(recurrent glioma)) OR TS=(glioma) | 111021 |
| #4 | #1 AND #2 AND #3 | 59 |
