# Supplementary material for: The effects of dabrafenib and/or trametinib treatment in Braf V600-mutant glioma: a systematic review and meta-analysis
Source: Neurosurg Rev. 2024 Aug 22;47(1):458. doi: 10.1007/s10143-024-02664-x (PMC11341626; doi:10.1007/s10143-024-02664-x)
Supplement: Supplementary file 3 — Supplementary file3 Supplementary Table 2.Publication bias and heterogeneity of summarized outcomes. (DOCX 13 KB) [file 10143_2024_2664_MOESM3_ESM.docx]

**Supplementary Table 2. Publication bias and heterogeneity of summarized outcomes**

| **Outcomes** | **Publication bias** | |
| --- | --- | --- |
|  | **Begg (*P* value)** | **Egger (*P* value)** |
| PFS | 0.602 | 0.992 |
| PFS rate | 0.117 | 0.371 |
| OS | 0.317 | NA |
| PR | 0.317 | NA |
| CR | 0.317 | NA |
| ORR | 0.317 | NA |
| RR | 0.851 | 0.145 |
| AEs | 0.652 | 0.056 |
| Death events | 0.317 | NA |

**Abbreviations:**NA:not available
